# The gating mechanism in cyclic nucleotide-gated ion channels

**DOI:** 10.1038/s41598-017-18499-0

**Published:** 2018-01-08

**Authors:** Monica Mazzolini, Manuel Arcangeletti, Arin Marchesi, Luisa M. R. Napolitano, Debora Grosa, Sourav Maity, Claudio Anselmi, Vincent Torre

**Affiliations:** 1 International School for Advanced Studies, Trieste, 34136 Italy; 2 INSERM U1006, Aix-Marseille Université, Parc Scientifique et Technologique de Luminy, Marseille, 13009 France; 3 Structural Biology Laboratory, Elettra-Sincrotrone Trieste S.C.p.A., Basovizza, Trieste, 34149 Italy; 4 National Heart, Lung and Blood Institute, National Institutes of Health, Bethesda, Maryland 20892 USA

## Abstract

Cyclic nucleotide-gated (CNG) channels mediate transduction in several sensory neurons. These channels use the free energy of CNs’ binding to open the pore, a process referred to as gating. CNG channels belong to the superfamily of voltage-gated channels, where the motion of the α-helix S6 controls gating in most of its members. To date, only the open, cGMP-bound, structure of a CNG channel has been determined at atomic resolution, which is inadequate to determine the molecular events underlying gating. By using electrophysiology, site-directed mutagenesis, chemical modification, and Single Molecule Force Spectroscopy, we demonstrate that opening of CNGA1 channels is initiated by the formation of salt bridges between residues in the C-linker and S5 helix. These events trigger conformational changes of the α-helix S5, transmitted to the P-helix and leading to channel opening. Therefore, the superfamily of voltage-gated channels shares a similar molecular architecture but has evolved divergent gating mechanisms.

## Introduction

Voltage-gated ion channels form a large super-family with a common overall architecture derived from a common ancestor^1^. This family includes usual voltage gated Na^+^, K^+^ and Ca^2+^ selective channels as well as other ion channels not directly gated by voltage, but by a variety of molecules and ions such as Ca^2+^, Na^+^ and cyclic nucleotide-gated (CNG) channels that are opened by the binding of cyclic nucleotides (CNs)^2–6^. Members of this family - voltage gated K^+^, Na^+^ and Ca^2+^ channels in particular - have been extensively studied and it is now well established that their gating is primarily controlled by the outward bending of the S6 α-helices^7,8^. According to this view, gating and permeation are independent processes with distinct structural and molecular basis. Influenced by the pivotal role of S6 in a number of K^+^ channels, early studies on CNG channels proposed that also in CNGA1 channels the gating was mediated by a similar motion of S6^9^. This hypothesis, however, was not confirmed by other experiments, which located the gate in the selectivity filter^10,11^. Thus, the same region of CNGA1 channels controls ion permeation and gating, leading to a more complex gating mechanism^12–15^.

Nevertheless, recent findings have suggested that in several types of K^+^ channels, such as BK, SK, MthK and K2P channels, gating is not primarily mediated by the bending of S6, but conformational rearrangements in the selectivity filter region^3,4,16–18^ play a major role. Therefore, the coupling between gating and permeation which is fundamental in CNG channels, is shared also by other members of the super-family.

CNG channels are formed by 4 subunits, either of type A (CNGA1, CNGA2, CNGA3, CNGA4 in mammals and CNGA5 in zebrafish) or of type B (CNGB1 and CNGB3)^6,19^. Native CNG channels in rods are composed of 3 CNGA1 and 1 CNGB1 subunits^20^, but homotetrameric CNGA1 channels are still functional and widely used as a native channel surrogate in functional and structural assays. The CNGA1 subunit from bovine rods is composed of 690 amino acids (a.a.)^21^; hydropathicity and biochemical analyses have identified six transmembrane (TM) α-helices referred to as S1 to S6 that are linked by non-spanning loops.

The structure determination of the eukaryotic CNG channels TAX-4 from *C*. *elegans* in the cGMP-bound open state by Cryo-EM at a resolution of 3.5 Å is a major contribution to the field^22–24^. The homology between the bovine CNGA1 channel − where the great majority of functional studies have been performed − and the TAX-4 channel is 60%. Further valuable information was brought also by the Cryo-EM structure of a prokaryotic CNG channel LliK in the presence of cAMP^23^. Despite this progress, to date, no high-resolution structure of a CNG channel in the closed and unliganded state is available, and therefore, the precise mechanism of ligand-mediated activation for this class of channels remains unknown.

To circumvent these difficulties, we took advantage of electrophysiology, site-directed mutagenesis, chemical modification and single molecule force spectroscopy (SMFS) to investigate the gating of CNGA1 channels embedded in a natural biological membrane. We introduced a single alanine or cysteine and pairs of cysteines in different domains with the intention to identify which domains move and determine their relative motion; indeed, if during gating pairs of exogenous cysteines come in close proximity (less than 10 Å), they will form an S-S bond and mutant channels could be locked in the closed or open state. We also used SMFS, which allows the identification of the interactions and relative motion between specific domains^25,26^. By means of this approach we identify conformational changes of S5 as an essential step for the gating of CNGA1 channels, similarly to what is likely to occur in K^+^ channels gated by intracellular ions such as Ca^2+^ and Na^+^^3,4^.

## Results

### Inactivation of mutant channels

Gating of CNG channels is initiated by the binding of CNs to the cyclic nucleotide-binding (CNB) domain and terminates with the opening of the gate located in the pore region, approximately some tens of Å apart^10,11^.

All native CNG channels from photoreceptors and olfactory sensory neurons - and the homotetrameric channels formed by the CNGA1 subunits - do not inactivate (i.e. no time dependent decrease of the current amplitude is observed) in the presence of a steady cyclic nucleotide concentration and physiological pH (Supplementary Fig. 1a)^27^. In order to locate key residues involved in the gating of CNGA1, we performed an extensive site-directed mutagenesis analysis with the aim to identify mutant channels with an unusual gating, such as inactivation.

**Figure 1:**
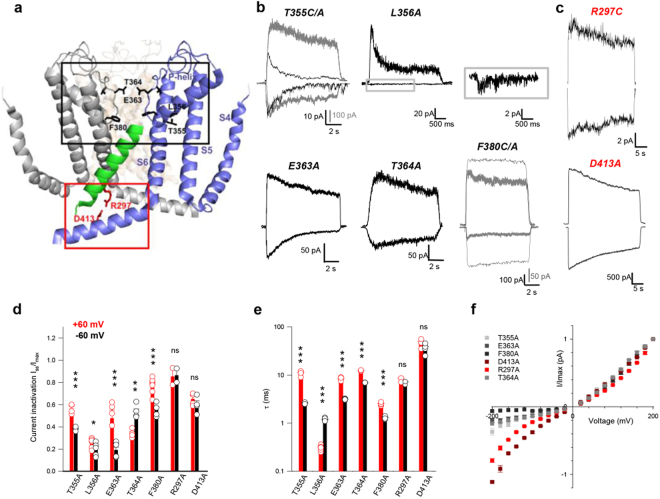
Inactivation of mutant channels. (**a**) Mapping CNGA1 residues on the TAX-4 structure (PDB ID: 5H3O): in grey and blue S4-S6, P-loop and C-linker regions of two opposite TAX-4 monomers; in green partial S5 region (S302-V317) of a third monomer. Key residues are in stick representation. Mutant channels that inactivate in a voltage dependent manner (black square); mutant channels that inactivate in a non-voltage dependent manner (red square). (**b**,**c**) Current recordings evoked by 1 mM cGMP, during voltage steps at ± 60 mV in symmetrical Na^+^ solution for mutant channels that inactivate in a voltage dependent manner (**b**) or that inactivate marginally (**c**). In panel (b) gray traces refer to T355A and F380A, respectively; trace at −60 mV for mutant channel L356A is enlarged in the grey box. (**d**) Decline of cGMP-activated current for inactivating mutant channels at ± 60 mV. Histograms summarize the average ± s.e.m. and circles indicate data from single experiments at +60 (in red) and −60mV (in black). *I*_ss_/*I*_max_ where *I*_max_ is the current amplitude immediately after the application of 1 mM cGMP and *I*_ss_ is the current at the steady state. (**e**) The time course of inactivation at +60 and −60 mV obtained by fitting with a single exponential (**d**). (**f**) Macroscopic *I–V* relationships obtained from current recordings as in Figure S2a scaled to the *I* flowing at +200 mV. Grey scale colors were used for mutant channels that inactivate in a voltage dependent manner and red scale colors for mutant channels that inactivate in a non-voltage dependent manner; R297A (n = 2), T355A ( = 10), L356A (n = 6), E363A (n = 11), T364A (n = 4), F380A (n = 5) and D413A (n = 4). Values represent average ± s.e.m. Unpaired two–tail T-test for T355A, L356A, E363A, T364A, F380A in d: t_6_ = −7,911, P < 0.001; t_10_ = −2,274; P < 0.05; t_8_ = 6,881, P < 0.001; t_6_ = −5,541; P < 0.01; t_10_ = 5,652; P < 0.001. Unpaired two–tail T-test for T355A, L356A, E363A, T364A, F380A in e: t_6_ = 12.981, P < 0.001; t_6_ = −24,02; P < 0.001; t_8_ = 19,01, P < 0.001; t_6_ = 41,021; P < 0.001; t_10_ = 14,985; P < 0.001. Further statistical tests for these mutant channels and for all other tested mutants are reported in Supplementary Table 2a,b. ns = not statistically significant (P > 0.05). (n.a.) represents mutant channels with experiments not available, (f.r.) represents mutant channels with no experiments due to fast and complete rundown of the current, (n.e.) represents mutant channels with no experiments because they did not express.

We mutated into cysteine and/or alanine residues R289 and P293 in the S4-S5 linker, from R297 to I308 in the S5 TM domain, from S320 to D328 in the extracellular end of the S5 TM domain and in the loop connecting S5 to the P-helix, from R345 to S371 in the pore region^10^, from F375 to S399 in the S6 TM domain^28^ from N400 to V424 in the C-linker^29^ and residues from L583 to N610, corresponding to the C-helix of the CNB domain^30^, (Fig. 1a, sequence alignment in Supplementary Table 1).

All tested mutant channels have an open probability (Po) close to 0 in the absence of cGMP and up to 0.8/0.9 in the presence of saturating concentration of cGMP, i.e. 1 mM. We have analyzed the selectivity properties of mutant channels near the filter region, such as T359A and T360A and some changes of selectivity were observed, but in agreement with the common view of these channels, i.e. to be poorly selective^13,31^. In some mutant channels in S5 and S6 we verified the ligand affinity, which was similar to WT CNGA1 channel. These mutant channels, however, differ in their expression level: some of them have a very high expression (up to 10 nA currents could be observed in excised patches) but in other mutant channels, such as R297C (Fig. 1c), the expression level is so low that recorded currents are dominated by channel fluctuations.

The characterization of cysteine and/or alanine mutants provides a systematic screening of the contribution of each residue to CNGA1 gating. Out of these 134 mutant channels, we found seven residues (R297A/C, T355A/C, L356A/C, E363A, T364A/C, F380A/C and D413A/C) that, in symmetrical Na^+^ conditions, exhibited inactivation (Fig. 1b,c and Supplementary Fig. 1a). Mutant channels E363C do not show any cGMP-activated current. In five of these mutant channels (T355A/C, L356A/C, E363A, T364A/C and F380A/C) gating at +60 mV was significantly different from that at −60 mV because the rate of decline of the cGMP-activated current depended on voltage and/or because the cGMP-activated current, measured at the steady state after inactivation, was voltage dependent (Fig. 1b–f, Supplementary Table 2a,b for statistical details). Inactivation of mutant channels R297A/C and D413A/C at +60 mV was not significantly different from that at −60 mV (Fig. 1d,e; P > 0,05, Supplementary Table 2a,b). At the steady state the I/V relations in mutant channels F380A were highly non-linear but not in mutant channels D413A (Fig. 1f and Supplementary Fig. 2a). In mutant channels T355A and F380A, following inactivation, Po was significantly larger at positive voltages (Supplementary Fig. 2b,c). Previously, we mutated several residues in the pore region and some of these mutant channels exhibited a voltage dependent inactivation that was reminiscent of the C-type inactivation present in the K^+^ channels^32^. During this kind of inactivation, residues in the extracellular vestibule have a significant conformational rearrangement: T364 in different subunits becomes closer and P366 becomes more accessible to extracellular reagents^32^.

Here we show the existence of two kinds of inactivation: one kind of inactivation exhibits a marked voltage dependency (Fig. 1b,d–f) and is observed when residues close to the pore region are mutated (Fig. 1a: T355, L356, E363, T364 and F380) and is similar to the C-type inactivation of K^+^ channels; the second kind of inactivation has an almost absent voltage dependency (Fig. 1c–f) and is observed when residues R297 and D413 - located at the interface between the transmembrane and cytoplasmic domain of CNGA1 channels (Fig. 1a) − are mutated. Therefore, the altered gating of mutant channels R297A/C, D413A/C could be caused by defects of the coupling between the binding of cGMP and channel opening. Residues R297 and D413 could be key players of the involved signal transduction mechanisms.

### The double mutant channel D413C_R297C can be locked in the open state

**Figure 2:**
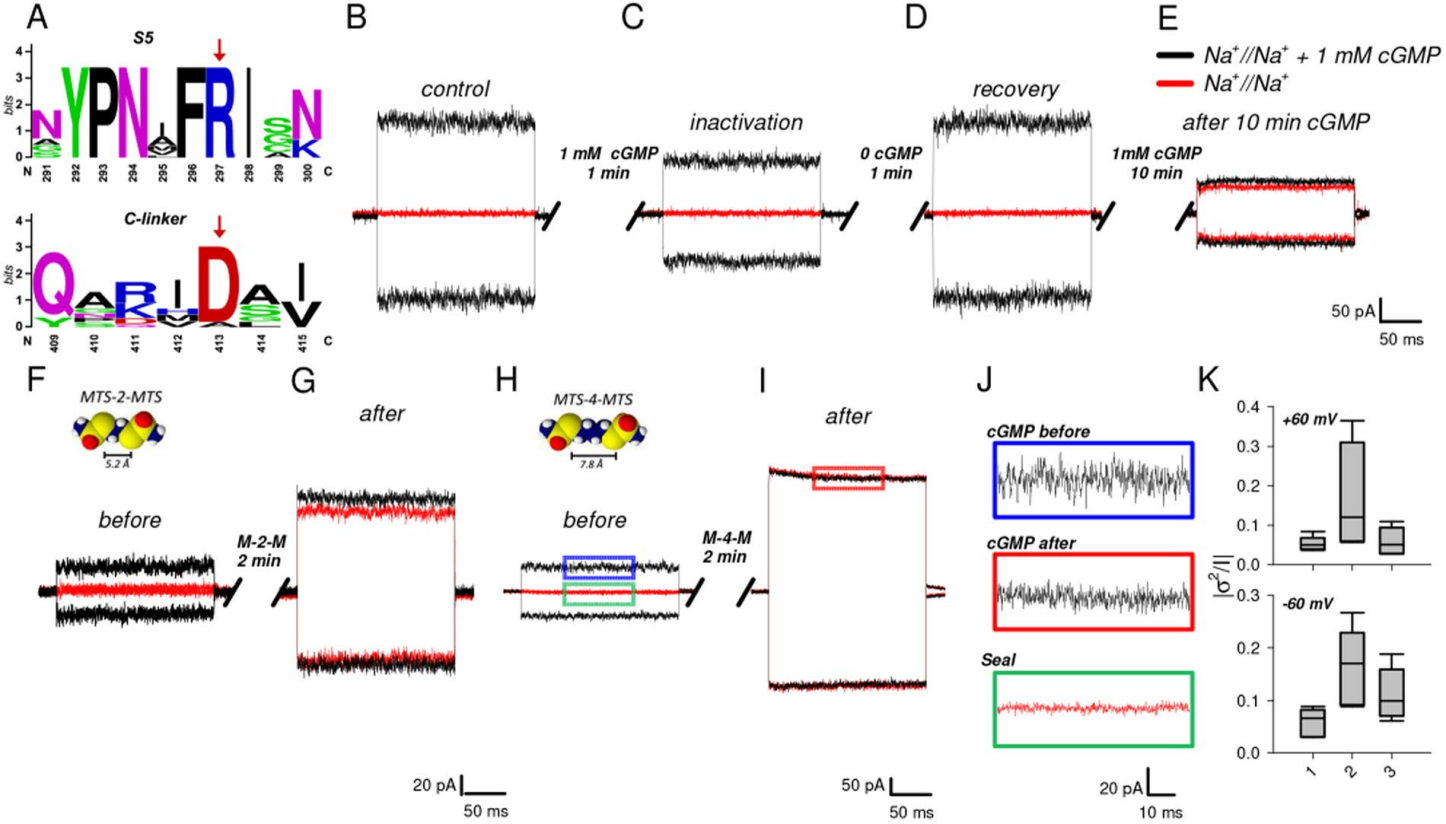
The double mutant channel D413C_R297C can be locked in the open state. (**A**) Web logo analysis of the sequence surrounding the conserved R297 and D413 residues (marked by an arrow). (**B**–**E**) Inactivation and locking in the open state of the double mutant channel D413C_R297C. in symmetrical Na^+^ solution in the presence of 1 mM cGMP at ± 60 mV immediately after the membrane patch excision (**B**) and after 1 minute (**C**). The recovery (**D**) of the current after 1 minute in the closed state. Irreversible lock of the current in the open state (**E**) after a prolonged exposure to cGMP (10 minutes). (**F**–**G**) MTS-2-MTS locking effect in the open state. cGMP-activated currents immediately after the membrane patch excision (**F**) and after 2 minutes of 100 μM MTS-2-MTS application in the open state (**G**). (**H**–**I**) As in **(F)** and (**G**), but in the presence of 100 μM MTS-4-MTS. Red/black lines represent the current evoked by 0/1 mM cGMP. (**J**) Enlargements of boxes in (**H**) and (**I**). (**K**) Box plot summarize the quantitative noise analysis in the absence (1: n = 6 at +60 mV and n = 6 at −60 mV) and in the presence of 1 mM cGMP, before (2: n = 6 at +60 mV and n = 6 at −60 mV) and after (3: n = 6 at +60 mV and n = 6 at −60 mV) application of MTS-4-MTS. The horizontal line within each box indicates the median of the data; boxes show the twenty-fifth and seventy-fifth percentiles of the data; whiskers show the fifth and ninety-fifth percentiles of the data.

R297 is highly conserved in all known CNG channels, whereas D413 is conserved in all CNGs with the exception of CNGA4, a subunit that fails to form functional channels when expressed in a heterologous system (Fig. 2A, Supplementary Table 1)^33^. Analysis of the Cryo-EM structure of TAX-4 channel captured in the open state (PDB ID:5H3O) located R297 in the intracellular portion of S5 (Fig. 1a)^22^. In order to verify the involvement of D413 and R297 in the gating process, we constructed the double mutant D413C_R297C. The mutant channel D413C_R297C inactivated, following the application of 1 mM cGMP. Within 1–2 minutes the cGMP-activated current was reduced by about 50% (Fig. 2B,C), but it recovered its original amplitude after cGMP removal (Fig. 2D, for details see figure legend), as in mutant channels D413A and R297A (Fig. 1c). Remarkably, we found that a longer exposure (about 10 minutes) to cGMP irreversibly locked the channel in the open state (Fig. 2E). The double mutant channel D413C_R297C was quickly and irreversibly locked in the open state by the simultaneous exposure to 1 mM cGMP and the cross-linkers MTS-2-MTS (Fig. 2F,G) or MTS-4-MTS (Fig. 2H,I); in this case, the current flowing through locked-open channels had an amplitude similar to and often larger than the cGMP-activated current before inactivation. Locking -in the open state - is associated to a potentiation and a strong reduction of the current fluctuations (Fig. 2J,K). In the locked open state, collected data indicate a reduction of the variance of current fluctuations σ^2^ of approximately 2 folds (please compare 2 and 3 in panel Fig. 2K). When channels fluctuate between an open and closed state σ^2^ is proportional to the closed probability P_c_ which is halved in the locked open state. In CNGA1 channels and in the presence of saturating cGMP concentration the P_o_ is close to 0.9^15^ and, therefore, in the locked open state P_o_ becomes close to 0.95. Analysis of the power spectrum of these fluctuations shows that the Lorentzian component with a cut-off frequency of around 1600 Hz is reduced when the electrostatic interactions between D413 and R297 are replaced by a S-S bond between residues in the same locations (Supplementary Fig. 3 and Fig. 2K), suggesting that fluctuations of the interactions between the C-linker and S5 are a source for the channel noise observed in saturating cGMP concentrations. SMFS experiments with the double mutant channel D413C_R297C in the presence of 1 mM cGMP indicated that an intrasubunit bond between the two exogenous cysteines can occur (Supplementary Fig. 4).

These experiments suggest that, when the electrostatic interactions between R297 and D413 are disrupted, CNGA1 channels inactivate, but when residues in position 297 and 413 are covalently linked by a S-S bond, CNGA1 channels do not inactivate and are locked in the open state. The next question is how do electrostatic interactions between R297 and D413 lead to the widening of the pore lumen? Two mechanisms could be in action: similarly to what occurs in usual voltage gated Na^+^ and K^+^ channels^22,23^ rearrangements of the S4-S5 linker and of the lower portion of S5 are transmitted to S6, and its motion and conformational changes are an essential component of the gating of CNGA1 channels. An alternative view is that the electrostatic interactions between R297 and D413 induce significant rearrangements of S5, which play a major role in the gating of CNGA1 channels, as opposed to what happens in Na^+^ and K^+^ channels.

### SMFS experiments show that S5 moves during gating and that loops change their folding

**Figure 3:**
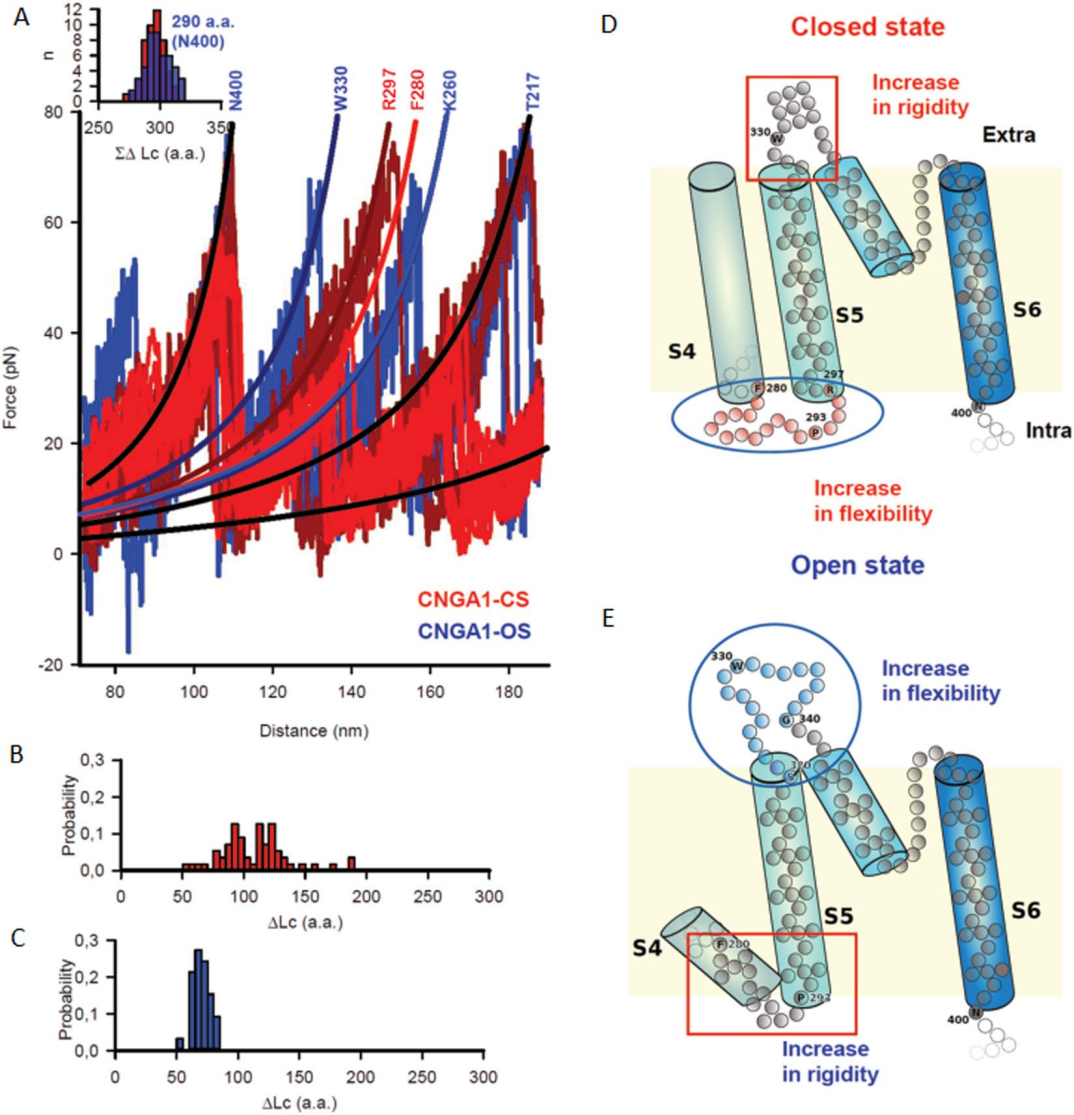
Flexibility in the loops between S4-S5 and S5-P-Helix during CNGA1 channels gating. (**A**) Superposition of F-D curves from unfolding of CNGA1 in the closed (n = 10) (CNGA1-CS, light and dark red) and open (n = 10) (CNGA1-OS, blue) states. The solid line represents the WLC fitting to the corresponding unfolded peaks, and the numbers represents the approximate peak position in the a.a. chain. Inset represents the ∑∆Lc histogram for the peak at N400 (290 a.a.), implying a conserved unfolding step for both open and closed states. (**B**) Distribution of unfolded length for the domains S6–S5 for CNGA1-CS (n = 110). (**C**) As in (**B**) but for CNGA1-OS (n = 71). (**D**–**E**) Cartoon representation of the S5-S6 domains, showing the change in flexibility between the intracellular and extracellular loops of S5 for closed and open state, respectively.

By using methods of SMFS, we unfolded CNGA1 channels from their C-terminal end^34^: the resulting F-D curves are composed of a series of force peaks, indicating that an unfolded region or a poorly structured polypeptide was in the process of being unfolded. During the unfolding of the transmembrane domain, a.a. were unfolded sequentially so that it was possible to identify the location of the unfolded region. The location of a force peak was obtained by fitting the experimental F-D curve with the Worm-Like Chain (WLC) model^35,36^, providing the values of the contour lengths (Lc). From the value of Lc it was possible to estimate the number of a.a. of the polypeptide unfolded between the occurrence of two consecutive force peaks (as ΔLc/0.4 nm)^36^. In CNGA1 channels the unfolding of the transmembrane domain occurs − both in the open and closed states - following the force peaks with an Lc of about 116 nm − corresponding approximately to N400 located at the base of S6 (Fig. 3A). In the closed state (red traces in Fig. 3A) there is a force peak corresponding to residues ranging from R297 to F280 which, in the open state (blue traces in Fig. 3A), splits into two force peaks corresponding approximately to W330 and K260. These changes indicate that in the closed state S5 is mechanically coupled to S6, but in the open state S5 is mechanically coupled to S4^34^. These data, in addition, also show major conformational changes during gating at the N- and C-terminal end of S5 as well as at the loops connecting S5 to the P-helix and S5 to the S4 helix. These changes can be deduced from the computation of the histograms of the increase of ΔLc from the force peak corresponding to N400 to the following force peak in the closed (Fig. 3B) and open states (Fig. 3C). In the closed state, the values of ΔLc ranged from 90 to 120 a.a. indicating that residues in the loop connecting S5 to the S4 - from approximately F280 to R297 - have a loose folding (Fig. 3D). In the open state, in contrast, the values of ΔLc ranged from approximately 60 to 80 a.a. so that the polypeptide chain from S320 to G340 in the extracellular loop, connecting S5 to the P-helix are poorly structured and can be unfolded with a low force (Fig. 3C). Therefore, during gating, major conformational changes occur primarily in S5 and in the connecting loops: in the open state the loop connecting S5 to the P-helix is loosely folded but the loop connecting S5 to S4 provides mechanical coupling (Fig. 3D,E).

An inspection of the Cryo-EM structure of TAX-4 channel (Supplementary Fig. 5) reveals that P293 is in the short loop connecting S4 to S5, where it acts as a helix initiator. Therefore, we constructed several mutant channels in position 293 with different intracellular helical-initiating inclination. Electrophysiological experiments indicate that all these mutant channels show an unusual gating and permeation which is more pronounced for the P293A mutant (Supplementary Fig. 6 and Supplementary Fig. 7). An altered gating and permeation caused by mutations at the intracellular side of S5 indicate that in the open state S5 is coupled to the pore walls and its conformational changes influence the filter structure. All together, these observations clearly suggest that gating is mediated by the coupling of molecular events at the N-terminal end of S5 to the selectivity filter.

### Cysteine scanning mutagenesis shows a state dependent accessibility of S5 and of the P-helix

**Figure 4:**
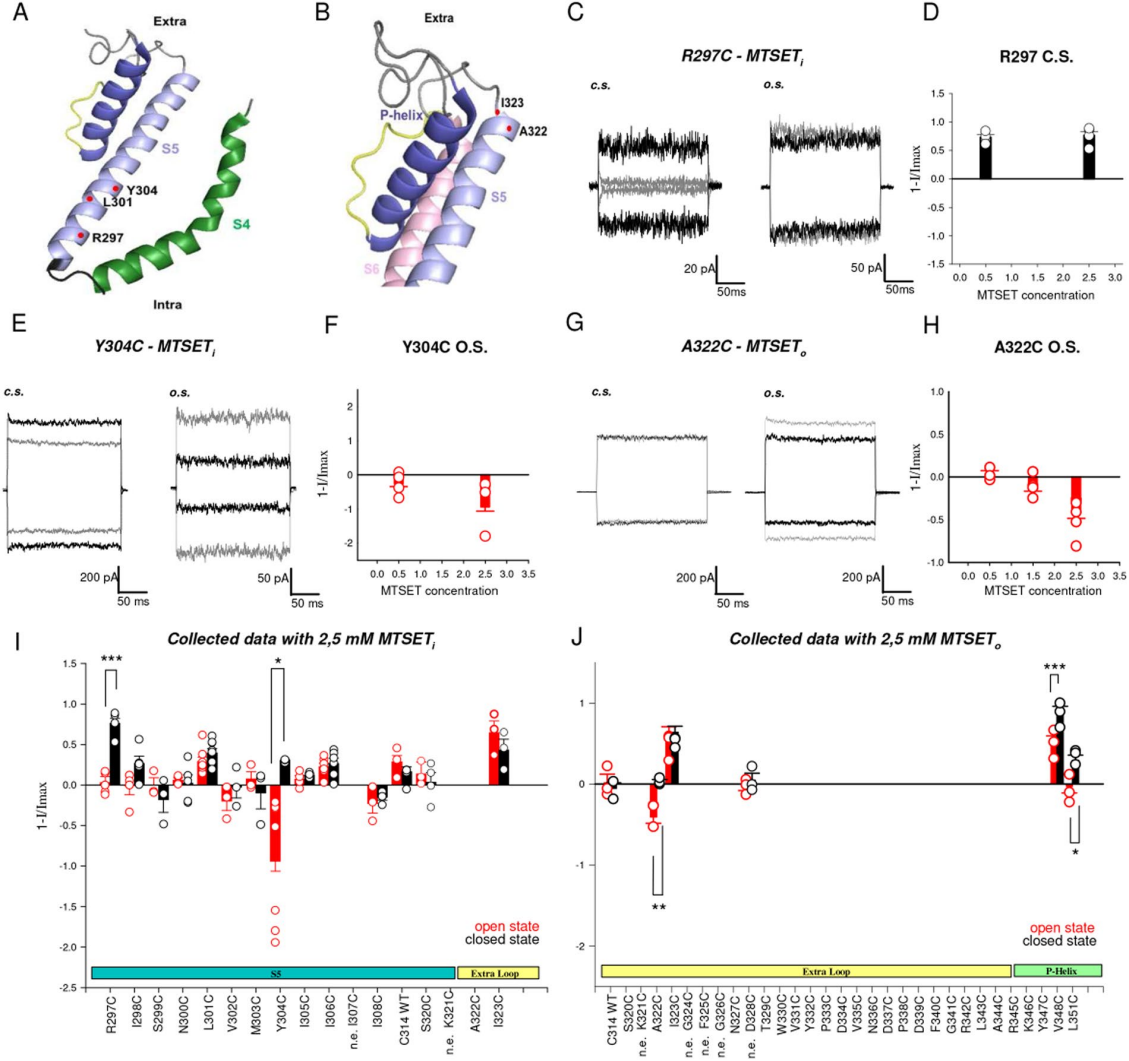
Cysteine scanning mutagenesis shows a state dependent accessibility of S5. (**A**) Mapping CNGA1 residues on TAX-4 structure (PDB ID: 5H3O) shows the location of key residues in the intracellular portion of S5. (**B**) as in (**A**) but for extracellular portion of S5. Key residues are highlighted with red spheres. Different regions (S4–S5 and P-helix) are illustrated in different colours. (**C**) Comparison between the cGMP-activated currents at ± 60 mV for the mutant channels R297C before (black) and after (grey) 3 minutes application of 2.5 mM MTSET to the intracellular side (*MTSET*_*i*_) in the closed (c.s.) and open (o.s.) states. (**D**) Plot representing the comparison of *MTSET*_*i*_ at different concentration (n = 4 for 0.5 mM and n = 5 for 2.5 mM) in the closed state for mutant channels R297C; histograms are the average ± s.e.m. and circles indicate data from single experiments. (**E**) as in (**C**) but for the mutant channels Y304C. (**F**) as in (**D**) but for the mutant channels Y304C in the open state (n = 4 for 0.5 mM and n = 7 for 2.5 mM). (**G**,**H**) as in (**E**) and (**F**) but for the mutant channels A322C with the addition of 2.5 mM MTSET to the extracellular side (*MTSET*_*o*_) and with different concentration of MTSET (n = 3 for 0.5 mM, n = 4 for 1.5 mM and n = 7 for 2.5 mM). (**I**,**J**) Histograms representing the average ± s.e.m. for many mutant channels, circles indicate data from single experiments after the application of 2.5 mM MTSET in the open (red) and closed (black) states. The number (n) of experiments for different mutant channels in different states varies between 3 and 8 (see also Supplementary Table 2). (n.e.) represents mutant channels without expression. Significativity is shown only for mutants where the MTSET effect was >30%. Unpaired two–tail T-test in i for R297C and Y304C: t_7_ = −7,529, P < 0.001; t_8_ = −2,649; P < 0.05. Unpaired two–tail T-test in j for A322C, V348C and L351C: t_8_ = −3,668, P < 0.01; t_12_ = −4,544, P < 0.001; t_6_ = −3,109, P < 0.05. Further statistical tests for these mutant channels and for all other tested mutants are reported in Supplementary Table 2c,d. 1-I/Imax represents the normalized current where I is the residual current measured in the presence of 1 mM cGMP after application of MTSET (in different conditions) and Imax is the cGMP-activated current at the beginning of the experiment.

In order to obtain further evidence of the pivotal role of S5 in the gating of CNGA1 channels, we then analyzed the conformational changes during gating of S5 and of the P-helix by Cysteine Scanning Mutagenesis (CSM). We scanned the residues in the intracellular (from R297 to I308) and extracellular portions (from S320 to D328) of S5 and in the P-helix (from R345 to S359) comparing the effect of MTSET in the closed and in the open states. According to TAX-4 structure residues R297, L301 and Y304 are located in the intracellular portion of S5 (Fig. 4A) and residues A322 and I323 are located at the extracellular end of S5 (Fig. 4B). As suggested by our SMFS experiments (Fig. 3) during gating, these residues are expected to undergo conformational changes. The cGMP-activated current for mutant channels R297C was abolished within 1–3 minutes, by MTSET in the closed state but not in the open state (Fig. 4C). Blockage by 0.5 and 2.5 mM MTSET had similar effects both in amplitude and time course (Fig. 4D), indicating that the residue in position 297 is easily accessible by intracellular MTSET. The effect of intracellular MTSET on mutant channels L301C was rather variable and collected data (n = 8 closed state; n = 8 open state) did not show a clear and significant state-dependent blockage by MTSET (Fig. 4I, Supplementary Table 2c). Mutant channels Y304C were potentiated by intracellular 2.5 mM MTSET in the open state, but not in the closed state (Fig. 4E). Potentiation caused by 0.5 mM MTSET was significantly lower than that observed with 2.5 mM (Fig. 4F), suggesting that residues in position 304 are less accessible than residues in position 297. Mutant channels A322C and I323C (Fig. 4B) had different reactivity to MTSET: mutant channel A322C was significantly potentiated by extracellular MTSET in the open but not in the closed state (Fig. 4G,J). Mutant channels I323C were blocked both in the open and closed states by extracellular but not by intracellular MTSET (Fig. 4I,J and Supplementary Table 2c,d). The effect of MTSET on the other tested mutant channels was either not state-dependent or could not be distinguished from what observed in the WT (Fig. 4I,J, - here named as C314 WT CNGA1 channels - Supplementary Fig. 1b–e, Supplementary Table 2c,d). The state-dependent effect of MTSET on mutant channels R297C, Y304C and A322C supports the notion that during gating there are conformational rearrangements − possibly associated to a motion of S5 related to the P-helix - involving the whole S5-helix. Indeed, according to the TAX-4 structure, R297, L301 and A322 span the entire S5 helix: these residues are located near the intracellular end, around the center, and at the C-terminal end of S5, respectively (Fig. 3A,B). The high density of hydrophobic residues in the extracellular portion of S5 and in the P-helix suggests that hydrophobic interactions could transmit the conformational rearrangements of S5 to the P-helix. Therefore, we performed a CSM also in the P-helix and we found 2 mutant channels V348C and L351C, which were blocked by extracellular MTSET in the closed, but not in the open state (Fig. 4J)^10^. Residues V348 and L351 are coupled to the pore walls and their motion is likely to cause the widening of the pore lumen.

The blockage and potentiation of MTSET reached a steady and complete effect within 5 minutes^28–30^ but with some consistent variability: in the closed state, blockage of mutant channels R297C occurred in less than 3 minutes and often was completed in 1–2 minutes but potentiation of mutant channels A322C, in the open state, required 3–5 minutes. These different action times are likely to be associated to the accessibility of mutated residues to MTSET^28–30^.

### The insertion of S-S bonds between S5 and the P-helix alters channel gating

**Figure 5:**
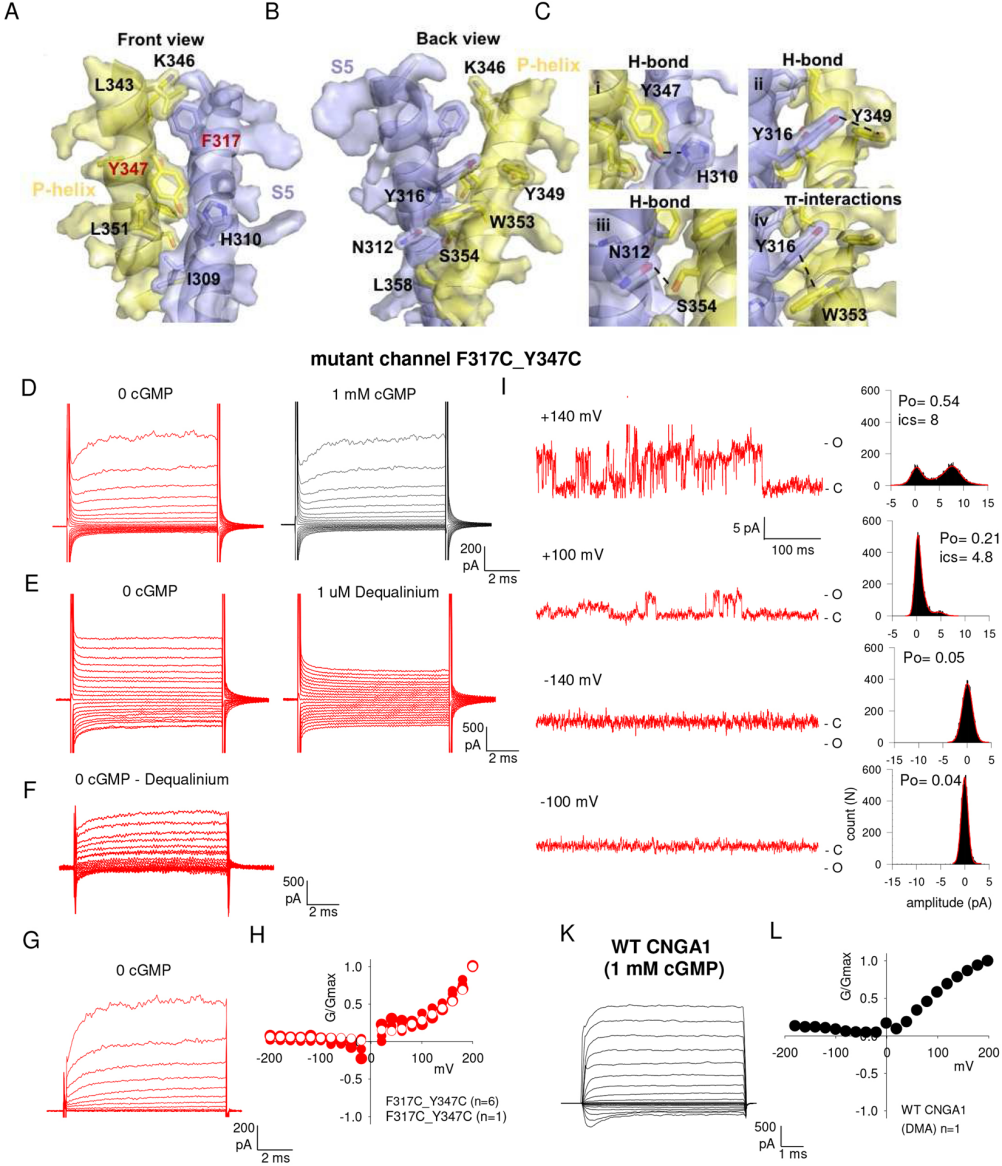
The double mutant channel F317C_Y347C is locked in the open state. (**A**,**B**) Mapping CNGA1 residues on TAX-4 structure reveals the presence of hydrophobic interactions. (**C**) Enlargement of panels a and b illustrating the network of H-bonds and hydrophobic π-interactions. (**D**) left: current recordings obtained in the absence of cGMP (0 cGMP) for mutant channels F317C_Y347C, elicited by voltage steps from −200 to +200 mV (Δ*V* = 20 mV); right: after the addition of 1 mM cGMP. (**E**) left: current recordings obtained in the absence of cGMP; right: 1 minute after the addition of 1 µM dequalinium. (**F**,**G**) Current recordings obtained in the absence of cGMP following subtraction of those currents recorded in the presence of dequalinium (**F**) or after P/-4 procedure for leak and capacitive artefact subtraction (**G**). (**H**) Dependence of G/Gmax on V with 1 µM dequalinium and in the absence of cGMP (n = 4, black) and in the presence of 1 mM cGMP (n = 3, red). (**I**) single channel recordings obtained in the absence of cGMP for mutant channels F317C_Y347C, elicited at different voltages. Amplitude histograms are shown at the right of each trace. isc, single-channel current amplitude. (**K**) current recordings obtained from the WT CNGA1 channels in the presence of 1 mM cGMP, elicited by the same voltage steps as in (**D**). (**L**) Dependence of G/Gmax on V obtained from the recordings of WT CNGA1 channels (**K**) in the presence (n = 1) of DMA.

We also constructed mutant channels with one exogenous cysteine in S5 and another cysteine in the P-helix, with the aim of establishing an S-S bond between S5 and the P-helix, possibly locking these mutant channels in the open state. Based on the high-resolution Cryo-EM structure of TAX-4 we built a model of the CNGA1 channel in the open state which shows that S5 and the P-helix are knitted together by a combination of an hydrogen-bonding network, with hydrophobic interactions involving Y316 (S5) and W353 (P-helix) (Fig. 5A–C). I309, H310, A313, Y316, F317 in S5 form a hydrophobic patch with L343, K346, Y347, L351, L358 in the P-helix that, together with the H-bonds, could be responsible for transmitting S5 conformational changes to the P-helix during ligand gating. These residues are highly conserved in all CNG channels, consistent with their key role in the coupling between S5 and the P-helix (Supplementary Table 1).

As F317 and Y347 could be two of the residues mediating the coupling between S5 and the P-helix, we constructed the double mutant channel F317C_Y347C in which a S-S bond was inserted between these two domains. When the mRNA for these double mutant channels was injected in some oocytes, no cGMP-activated current was recorded but the I/V relations measured in excised patches - in the absence of cGMP - were outwardly rectifying (Fig. 5D,E). This result is in contrast with what is usually recorded in uninjected oocytes (Supplementary Fig. 8a,b) suggesting that the double mutant channels F317C_Y347C could have some unusual properties and could be locked in the open state.

To verify whether these mutant CNGA1 channels were locked in the open state, we added 1 µM of Dequalinium to the intracellular medium, a powerful and specific blocker of CNGA1 channels^37^: after addition of Dequalinium, the I-V relations became linear within 60 seconds (Fig. 5E) indicating that the F317C_Y347C mutant channels were locked in the open state, as also demonstrated by the application of DTT (Supplementary Fig. 8f). The Dequalinium sensitive current, obtained as the difference of the current initially measured either in the presence (Fig. 5E, left panel) or absence of cGMP minus the current measured in the presence of Dequalinium (Fig. 5E, right panel), was highly nonlinear (Fig. 5F). In some experiments (Fig. 5G) we recorded a rectifying current in which at positive voltages the current clearly increased with a time constant of about 1 ms. The relation between G/Gmax (Fig. 5H) of the Dequalinium sensitive current showed a significant voltage dependency. The voltage dependence of current recordings (Fig. 5G,H) obtained from the mutant channel F317C_Y347C in the absence of cGMP is reminiscent of what observed in some kind of K2P K^+^ channels, such as the TRAAK and TREK-1 channels and could originate, partially, from similar mechanisms^18^. In TRAAK and TREK-1 channels the voltage dependent gating is attributed to the simultaneous presence of three or four ions inside the selectivity filter, whereas, in CNG channels − because of the shorter size − only two ions are likely to be simultaneously present in the pore^18^.

We recorded also single channel openings at different voltages (Fig. 5I) and the values of the single channel current (isc) were 8 and 4.8 pA and the Po was 0.54 and 0.21 at the membrane voltages of +140 and +100 mV, respectively. The dependence of G/Gmax of the double mutant channel F317C_Y347C (Fig. 5H) locked in the open state was very similar to what seen in the WT CNGA1 channels in the presence of large organic cations such as dymethylammonium (Fig. 5K,L) or etylammonium^14^. The similarity suggests a common molecular mechanism, i.e. the motion of the voltage sensor S4 and its coupling to S5. Comparable results were also obtained with the double mutant channels W330C_Y347C, but in this case the I/V relations of the Dequalinium sensitive current were less rectifying (Supplementary Fig. 8c–e).

Overall our CSM analysis clearly indicates that the S5-helix undergoes significant conformational changes during gating. When an S-S bond between S5 and the P-helix is inserted, the mutant channel is locked in the open state and becomes voltage gated, similarly to what observed in WT CNGA1 channels in the presence of large organic cations^13,14^.

### Conformational changes of S6 and of the C-linker

**Figure 6:**
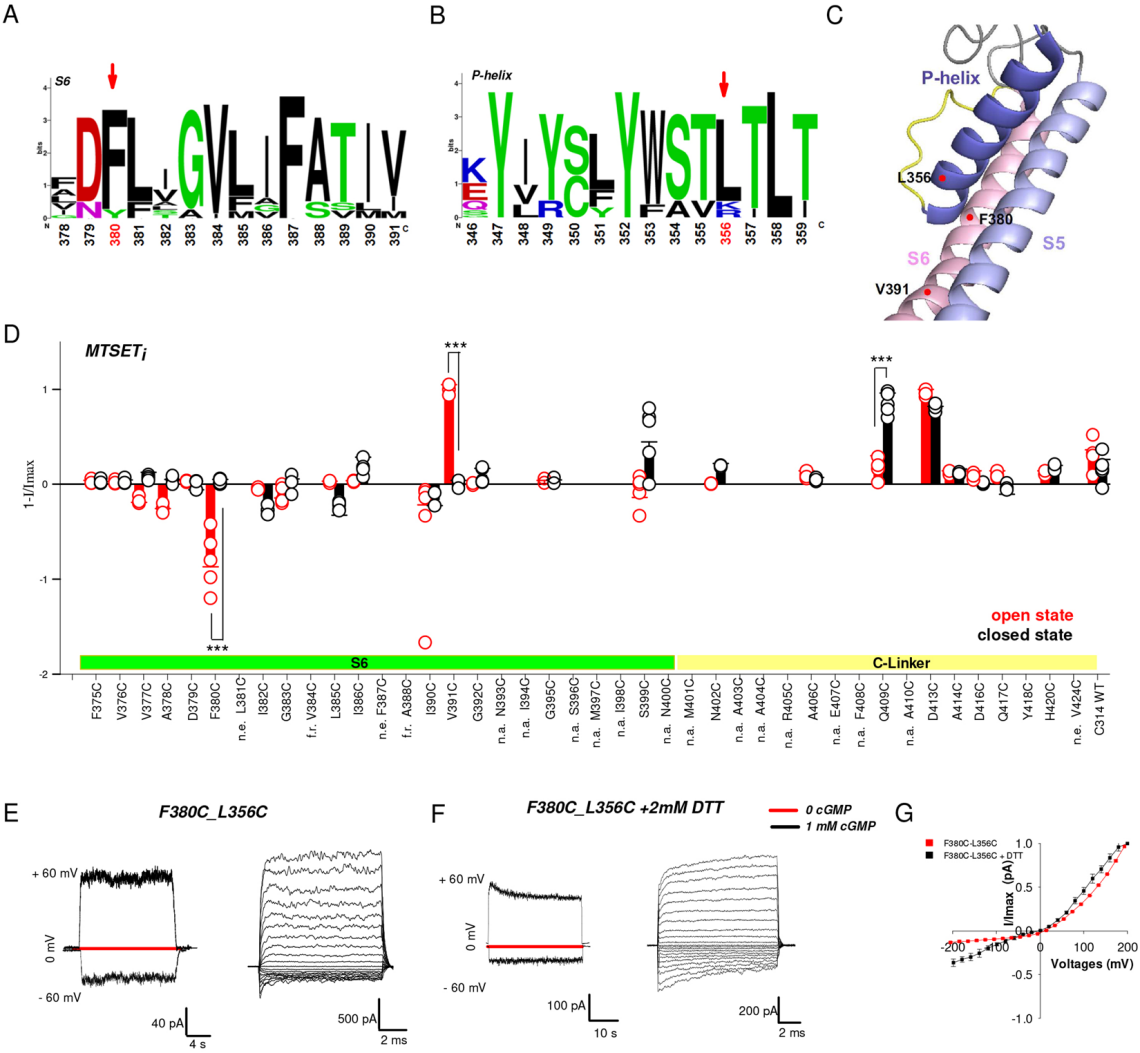
Cysteine scanning mutagenesis in S6 and C-linker and S-S bridges between S6 and the P-helix in the open state. (**A**,**B**) Web logo analysis of S6 (**A**) and P-helix (**B**) in CNG channels based on the alignment given in Supplementary Table S1. (**C**) Mapping CNGA1 residues on TAX-4 structure (PDB ID: 5H3O) shows the location of mutated residues in S6 and the P-helix. (**D**) Collected data of the effect of 2.5 mM MTSET_i_ in the open (red) and closed (black) state in the S6 transmembrane domain and in the C-linker. (**E**) left: current recordings obtained in the absence (red) and in the presence of 1 mM cGMP (black) at ± 60 mV for mutant channels F380C_L356C. cGMP was added some seconds after setting the voltage command at ± 60 mV. The double mutant does not inactivate and it is gated by cGMP. Right: current recording obtained in the presence of 1 mM cGMP for mutant channels F380C_L356C, elicited by voltage steps from −200 to +200 mV. (**F**) as in (**E**) but in the presence of 2 mM DTT present in the patch pipette. (**G**) Macroscopic *I–V* relationships of F380C_L356C mutant channels in the presence (n = 3) and in the absence (n = 4) of DTT. The number (n) of experiments for different mutant channels in different states varies between 3 and 8 (for details see also Supplementary Table 2). (n.e.) represents mutant channels without expression, (n.a.) represents mutant channels with no experiments where the expression was too low. (f.r.) represents mutant channels with no experiments where the run-down was too fast. Significativity is shown only for mutants where the MTSET effect was >30%. Unpaired two–tail T-test in d for F380C and V391C: t_10_ = −7,407, P < 0.001; t_4_ = 25,158. Further statistical tests for these mutant channels and for all other tested mutants are reported in Supplementary Table 2e.

We also performed a complete CSM of residues from F375 to V424 corresponding to S6 (from F375 to S399, Fig. 6A,C,D) and the portion of the C-linker – referred to as the A’ helix composed of about 20 a.a. – containing the key residue D413 (Fig. 1A, Fig. 2A, Supplementary Table 1)^38^. If in the open state D413 approaches R297 in the intracellular portion of S5 (Fig. 2), some conformational changes are also expected to occur also in the C-linker and S6, the contiguous region in the primary structure (Supplementary Table 1). Out of the mutant channels from F375C to S399C we found two mutant channels which reacted to MTSET in a state-dependent manner: F380C (Fig. 6D, Supplementary Table 2e) was potentiated by MTSET in the open but not in the closed state and V391C (Fig. 6D, Supplementary Table 2E) was abolished by MTSET in the open but not in the closed state. The remaining mutant channels (Fig. 6D, Supplementary Table 2e) reacted to MTSET similarly to the WT (in Fig. 4 named C314 WT CNGA1 channels) or the effect was below the 30% threshold (see Data Analysis subheading in the experimental procedures). Mutant channels V384C and A388C had a quick rundown and their reaction to MTSET could not be determined (Fig. 6D). Mutant channels S399C (Fig. 6D) had a variable run down that developed in 2–5 minutes^28,39^. Some mutant channels (Fig. 6D) did not provide functional channels gated by cGMP.

According to the TAX-4 structure, V391 is near the intracellular end of the S6 and F380 is in the extracellular portion of S6 at a close distance from the P-helix (Fig. 6A–C) and in particular from L356. Therefore, conformational changes of residues in position F380 could be coupled to rearrangements of the P-helix and underlying gating. In order to test this possibility, we constructed the double mutant channel F380C_L356C with an S-S bond between S6 and the P-helix (Fig. 6C). If the motion of S6 and in particular of F380 is pivotal in gating, these mutant channels should be either locked in the open or closed state and will not be gated by cGMP. Mutant channels F380C_L356C, in contrast, open only in the presence of cGMP and cannot be locked in the open state (Fig. 6E) as the double mutant channel D413C_R297C, but exhibit a strong voltage dependent gating (Fig. 6E,G). The presence of the S-S bond was verified by adding 2 mM of DTT to the extracellular side (Fig. 6F) of the membrane^29^. When the S-S bond was disrupted by DTT, the double mutant channels F380C_L356C inactivated (Fig. 6F) during a long exposure to cGMP, with a time course intermediate to those observed in the single mutant channels F380C and L356C (Fig. 1) and their I-V relations were less rectifying (Fig. 6E–G). Therefore, we conclude that the conformational changes of S6 are not crucial events occurring during gating of CNGA1 channels.

**Figure 7:**
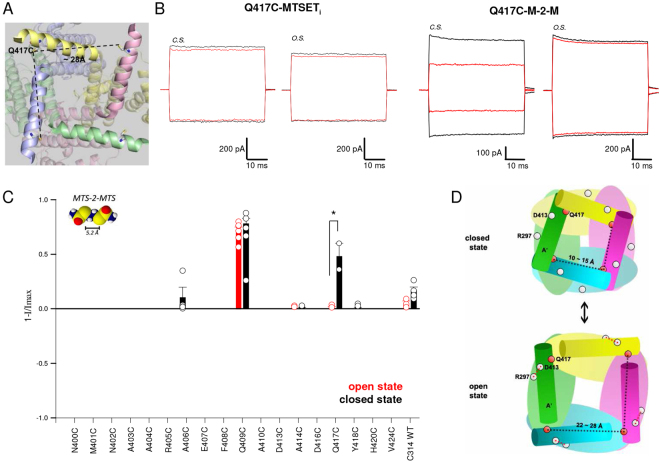
Cysteine scanning mutagenesis in S6 and analysis of M-2-M compound in the open and closed states. (**A**) Mapping of Q417C mutant channel on TAX-4 structure (PDB ID: 5H3O): its distance between adjacent subunits is more than 25 Å apart. (**B**) left: the cGMP-activated currents at ± 60 mV for the mutant channels Q417C before (black) and after (red) 5 minutes application of 2.5 mM MTSET to the intracellular side (*MTSET*_*i*_) in the closed (c.s.) and open (o.s.) states. Right: the cGMP-activated currents at ± 60 mV for the mutant channels Q417C before (black) and after (red) 5 minutes application of 100 µM M-2-M in the closed and open states. (**C**) Histograms representing the average ± s.e.m, after the application of 100 µM M-2-M in the open (red) and closed (black) states. Significativity is shown only for mutants where the M-2-M effect was >30%. Unpaired two–tail T-test for Q409C: t_8_ −9,907; P < 0.001. Unpaired two–tail T-test for Q417C: t_12_ = −6,57, P < 0.05. Further statistical tests for these mutant channels and for all other tested mutants are reported in Supplementary Table 2f. 1-I/Imax represents the normalized current where I is the residual current measured in the presence of 1 mM cGMP after the application of M-2-M and Imax is the cGMP activated current at the beginning of the experiment. (**D**) Schematic representation of mutant channels Q417C highlighting that residues Q417 belonging to adjacent subunits have a different distance in the closed and open states.

We also scanned residues in the A’ helix of the C-linker from N400 to V424 by CSM. Mutant channels D413C (Fig. 6D) were abolished by MTSET both in the open and closed states (Supplementary Table 2e) and mutant channels Q409C (Fig. 6D, Supplementary Fig. 9) reacted to MTSET in a state-dependent manner and were abolished in the closed but not in the open state. If the C-linker of CNGA1 channels is homologous to the solved structure of the C-linker of TAX-4 the residues in the A’ helix of homologous subunits are near in the open state and possibly at a distance which is state-dependent. In order to verify whether during gating there is a change of the distance among the A’ helices in different subunits, we tested the effect of cross-linkers of different length on those mutant channels not blocked by MTSET (Fig. 6D). Mutant channels Q417C were not blocked by MTSET neither in the closed or open states (Fig. 7B,C and Supplementary Table 2e,f) but were blocked by the cross-linkers M-2-M in the closed but not in the open state (Fig. 7B,C, Supplementary Table 2e,f). These results suggest that residues in the A’ helix of the C-linker in proximity to Q417 are near each other in the closed state and move apart in the open state, so that D413 approaches R297 in S5 initiating gating of CNGA1 channels (Fig. 7D). This hypothesis is confirmed by the TAX-4 structure in the open state where Q417 belonging to adjacent subunits in the tetramer are more than 25 Å apart (Fig. 7A). Blockage of mutant channels Q417C by M-2-M in the closed state suggests that in this conformation residues Q417 in adjacent subunits are at a distance that is below 20 Å.

## Discussion

The proposed mechanism of gating is based on the homology of the CNGA1 channel with the reported structure of the TAX4 channel in the open state^22^ and on the mutagenesis of the CNGA1 channel in the closed and open states. Therefore, our model of gating is not based on the comparison of a resolved molecular structure in the open and closed states, but on a detailed and extensive functional investigation, supported by the homology with the TAX4 structure^22^ in the open state. The major conclusion of our electrophysiological and SMFS experiments is that – during gating - the signal transmitted from the CNBD to the gate located in the filter region^11^ is not an outward bending of the intracellular portion of S6, but involves conformational changes of S5, in contrast with what is the usual thought^9^ and has been recently assumed^22^. Let us, now, recapitulate the experimental results at the basis of the proposed model of gating.

Firstly, CSM of S5 has shown that MTSET had a state-dependent effect on several mutant channels (Fig. 4) encompassing S5 throughout its length. CSM and SMFS experiments (Fig. 4, Fig. 3) show that during gating there are conformational changes of S5, in the extracellular loops connecting S5 to the P-helix and in the intracellular loops connecting S4 to S5. Our results are consistent with the recently solved structure of the CNG channel in the open state from the *C*. *elegans*, where residue R308 (equivalent to our R297) in the S5-helix and D429 (equivalent to D413) in the A’-helix are involved in a salt bridge interaction, coupling conformational changes in the CNBD to channel gating similarly – to some extent - to what observed in HCN channels^40^. Hydrophobic interactions between residues in the upper portion of S5 and in the P-helix have a major role in a way reminiscent of what occurs in the Slo2.1 channels^4,41,42^ and in the Ca^2+^-activated potassium channels KCa3.1^3^.

Secondly, we have introduced exogenous cysteines in distant domains of CNGA1 so to identify key events underlying gating. The analysis of three double mutants reveals these key events: (1) when residues in position D413 - in the C-linker – and in position R297 – in the intracellular portion of S5 – are cross-linked (Fig. 2), mutant channels R297C_D413C channels are locked in the open state; (2) when the relative motion between S6 and the pore region is drastically reduced and possibly abolished, gating of mutant channels F380C_L356C is only marginally affected, indicating that conformational changes of S6 are not pivotal in the gating of CNGA1 channels; (3) when an S-S bond between S5 and the P-helix is inserted, mutant channels F317C_Y347C are locked in the open state (Fig. 5).

**Figure 8:**
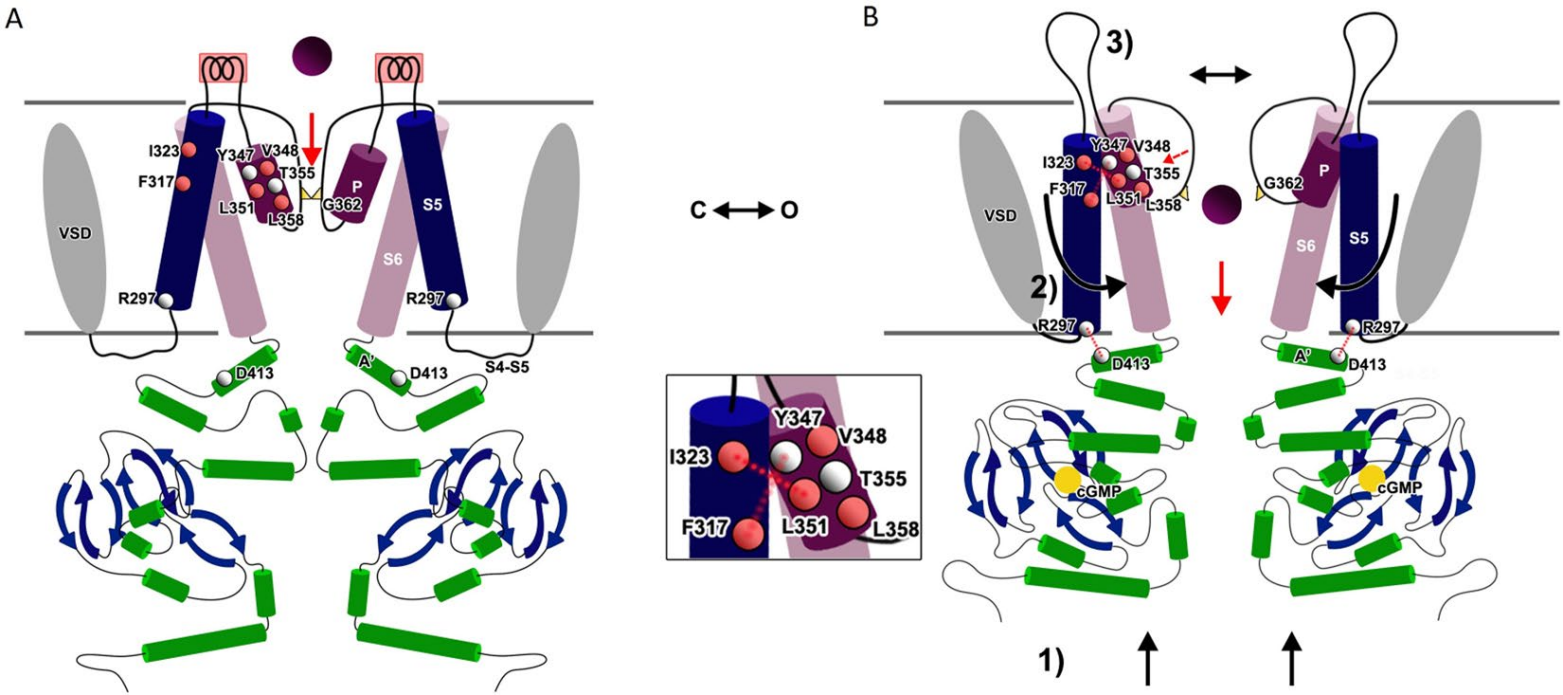
Proposed gating mechanism. (**A**,**B**) Cartoon representation of the proposed gating mechanism of CNGA1 from the closed state (**A**) to the open state (**B**). Before the binding of CNs, the CNBD is unstructured and D413 and R297 do not interact (**A**). Upon CNs binding the CNBDs change their conformation and move toward the transmembrane domain (1). The C-linker is then pushed toward the channel core. The interaction between R297 in the intracellular part of S5 transmembrane domain and D413 in the C-linker causes the motion of S5 so that the P-helix can interact with S5 (2). Opening of the gate at the selectivity filter (3). Sequence tracts from S4-S5 to S5 are coloured in blue, S6 in pink, P helices are coloured in magenta. Key residues are highlighted: hydrophobic residues are coloured in red, other residues are grey.

Inspired also by previous observations^43–45^, we propose that the coupling between the binding of CNs to the conformational changes in the transmembrane domain occurs following these key steps: i- the binding of CNs is followed by rearrangements in the C-linker with the A’ helices of neighboring subunits that move apart from each other (Fig. 7D, Fig. 8); ii- then, the movement of residues D413 in the A’ helices towards R297 in S5 favors electrostatic interactions between these residues (Fig. 7D, Fig. 8); iii - these electrostatic interactions induce conformational changes primarily in S5 (Figs 3–6); iv – these conformational changes are transmitted to the P-helix by hydrophobic interactions between S5 (I309, H310, Y316, F317) and the P-helix (L343, Y347, L351, L356 and L358); v – conformational changes in the P-helix convert the binding of cGMP in the widening of the pore lumen (Fig. 8B). Accessibility experiments show that residues E363, T364 and P366 are accessible only from the extracellular side, while residues T360 and I361 are accessible to intracellular reagents^10^. Therefore, the gate corresponds to residue G362^11^.

The key residues R297, V348, L351, L356, A322, I323, F380 and D413 of bovine CNGA1 channels examined in this work are highly conserved among the CNG channel family (Supplementary Table 1), and therefore we expect the molecular mechanisms underlying gating of bovine CNGA1 channels to be common to all CNG channels.

We conclude that the superfamily of voltage gated channels shares a common architecture, but within this large family the gating mechanisms that many types of channels adopt are different^46^. We propose that ion channels of the superfamily of voltage gated channels opened by the binding of CNs have a gating mechanism where the motion of S6 is not the main gate: in CNG channels the signal coupling the binding of CNs to the channel opening flows primarily along S5 and the motion of S6 is not the essential step during gating.

## Methods

### Ethical approval

All studies were approved by the SISSA’s Ethics Committee according to the Italian and European guidelines for animal care (d.l.26, March 4th 2014 related to 2010/63/UE and d.l. 116/92; 86/609/C.E.). Oocytes were harvested from female *Xenopus laevis* frogs (‘Xenopus express’ Ancienne Ecole de Vernassal, Le Bourg 43270, Vernassal, Haute-Loire, France) using an aseptic technique or, if necessary, purchased from Ecocyte Bioscience (Am Fӧrderturm, 44575, Castrop-Rauxel, Germany). *Xenopus laevis* were kept in tanks – usually 6–8 animals per tank – and were exposed to a 12/12 hours dark/light cycle. All *Xenopus laevis* surgeries were performed under general anesthesia, obtained by immersion in a 0.2% solution of tricaine methane sulfonate (MS-222) adjusted to pH 7.4 for 15–20 min. Depth of anesthesia was assessed by loss of the righting reflex and loss of withdrawal reflex to a toe pinch. After surgery, animals were singly housed for 48 h. Frogs were monitored daily for 1 week postoperatively to ensure the absence of any surgery-related stress. Postoperative analgesics were not routinely used. Considering the simplicity of the procedure, the lack of complications, the effectiveness of anesthetic regimen and the reduction in the number of animals likely to be used compared to the number that would be required if only one surgery were permitted, multiple surgeries on a single animal were performed. Individual donors were used up to five times, conditional upon the health of an individual animal. Recovery time between oocyte collections from the same animal was maximized by rotation of the frogs being used. A minimum recovery period of 1 month was ensured between ovarian lobe resection from the same animal to avoid distress. Evidence of surgery-related stress resulted in an extended rest period based on recommendations from the veterinary staff. After the fifth terminal surgery frogs were humanely killed through anesthesia overdose via 2 h of immersion in a 5 g/l MS-222 solution adjusted to pH 7.4.

### Molecular biology

The CNGA1 channel from bovine rods consisting of 690 a.a. was used^21^. Selected residues were replaced as previously described^10^ using the Quick Change Site-Directed Mutagenesis kit (Stratagene, La Jolla, CA, USA) or ordered from Genscript, 860 Centennial Ave., Piscataway, NJ 08854, USA. Point mutations were confirmed by sequencing, using a LI-COR sequencer (4000 l; LI-COR Biosciences, Lincoln, NE, USA). cDNAs were linearized and were transcribed to cRNA *in vitro* using the mMessage mMachine kit (Ambion, Austin, TX, USA).

### Oocyte preparation and chemicals

Mutant channel cRNAs were injected into *Xenopus laevis* oocytes. Oocytes were prepared as previously described^10^. Injected eggs were maintained at 18 °C in a Barth solution supplemented with 50 μg/ml gentamycin sulfate and containing (in mM): 88 NaCl, 1 KCl, 0.82 MgSO_4_, 0.33 Ca(NO_3_)_2_, 0.41 CaCl_2_, 2.4 NaHCO_3_ and 5 Tris-HCl, pH 7.4 (buffered with NaOH). During the experiments, oocytes were kept in a Ringer solution containing (in mM): 110 NaCl, 2.5 KCl, 1 CaCl_2_, 1.6 MgCl_2_ and 10 Hepes, pH 7.4 (buffered with NaOH). Usual salts and reagents were purchased from Sigma Chemicals (St Louis, MO, USA).

### Application of sulfhydryl-specific reagents

Sulfhydryl-specific reagents effect was tested using a concentration of 0.5, 1.5 and 2.5 mM. MTSET links covalently to only one cysteine. Cross-linker compounds such as MTS–2–MTS (1,2-Ethanediyl bismethanethiosulfonate) and MTS–4–MTS (1,4-Butanediyl bismethanethiosulfonate) had different maximum cross-linking spans — i.e. the longest distance between the S atoms of the cross-linker reacting with the S atoms of cysteine and forming S–S bonds^47^. These compounds, in contrast, could link to two cysteines. The cross-linker MTS–2–MTS had a cross-linking span of 5.2 Å while MTS–4–MTS had a cross-linking span of 7.8 Å. The different effect of reagents able to link to one cysteine - such as MTSET, MTSPT, MTSPtrEA - and those able to link to two cysteines - M-X-M cross-linkers - with the same size and volume was used to discriminate whether blockage was caused by bridging two cysteines by the binding of the compound to one end only. MTS compounds were purchased from TRC (Toronto Research Chemicals, Canada). In some selected experiments, the application, in the bath or in patch pipette solution, of 2 mM dithiothreitol (DTT) was used to reduce the formation of disulfide bonds^29^.

### Recording apparatus

cGMP-gated currents from excised patches were recorded with a patch-clamp amplifier (Axopatch 200; Axon Instruments Inc., Foster City, CA, USA), 2–6 days after RNA injection, at room temperature (20–24 °C)^30^. The perfusion system allowed a complete solution change in less than 0.1 seconds^10,27^. Macroscopic and single-channel current recordings were obtained with borosilicate glass pipettes which had resistances of 2–5 MOhm in symmetrical standard solution. The standard solution on both sides of the membrane consisted of (in mM) 110 NaCl, 10 Hepes and 0.2 EDTA (pH 7.4). Solutions were buffered with tetramethylammonium hydroxide at the desired pH. When the cation X^+^ was used as the charge carrier, NaCl in the standard solution on both sides of the membrane patch was replaced by an equimolar amount of the cation X^+^. We used Clampex version 10.0 for data acquisition. Recordings to perform noise analysis were low-pass filtered at 10 kHz. All other macroscopic current recordings were low-pass filtered between 1 to 10 kHz.

### Data analysis

Single-channel currents (i) were estimated from patches containing only one channel and fitting normalized all-point histograms with two-component Gaussian functions. Data in this manuscript are presented as mean ± SEM (V-I relationships), dot-plots (with means ± SEM) or box-and-whisker when data sets need to be statistically compared. n indicates the number of patches.

Data analysis and figures were made with Clampfit version 10.1 (Molecular Devices, Sunnyvale, CA, USA) and Sigmaplot 12.5 (Systat Software, Chicago, IL, USA).

We excluded data when we lost the patch during the experiments, when the level of expression was too low and we could not distinguish the noise from random channel openings, from electrical noise due to an unstable patch and/or from spurious electrical noise. We kept only data obtained during experiments in which the amplitude of the seal (i.e. the current evoked by voltage pulses in the absence of cGMP) was stable. These criteria were not used for those experiments describing the locking of channels in the open state (see Fig. 2). All these criteria were established prior to data collection.

Statistical significance for parametric analysis was determined using unpaired two-tailed T-test or single-variable ANOVA, as specified in Supplementary table 2. For pairwise comparisons, a Holm–Sidak test was used following ANOVA. P < 0.001 (***), P < 0.01 (**) and P < 0.05 (*) were considered significant, as indicated. No statistical methods were used to predetermine sample sizes, but our sample sizes are similar to those reported in previous publications^28–30,32^. In this study particular care was given to MTSET/MTS-2-MTS reactivity experiment interpretation, as “run-ups” attributed to de-phosphorylation of the channel by endogenous patch-associated phosphatases are known to occur in CNGA1 channels^48^. Indeed, we often observed a slow time-dependent “run-up” or “run-down” of the cGMP-gated current (after 5 minutes, up to 10% of the current initially recorded) in control conditions. Therefore, to avoid that such non-specific effect might be attributed to MTS reactivity, in this study only changes (either current potentiation or blockage) greater than 30% were considered. These effects are usually highly significant (P < 0.001, see Figs 4, 6, 7).

### SMFS experiments and data processing

The NanoWizard 3 AFM system (JPK) and an inverted optical microscope (Olympus IX71) were used under liquid conditions in Standard solution (in mM: 110 NaCl, 10 HEPES and 0.2 EDTA pH 7.4 buffered with NaOH) with or without 2 mM cGMP. Rectangular silicon nitrite gold-coated cantilevers (HYDRA2R-50NGG from APPNANO) were used to localize plasma membrane patches and to perform SMFS experiments. SMFS experiments were performed using membrane extracted from injected oocytes expressing constructs (P293A and D413C-R297C) at a high level. Oocytes were attached to a mica substrate and clean fragments of membrane remained anchored to the substrate with the intracellular side exposed to the bathing medium and to the cantilever tip of the AFM. We used the worm-like chain (WLC) model where Lp is the persistence length (equal to 0.4 nm), a parameter that represents the stiffness of the molecule. For each tip-sample separation (D or TSS) value, the WLC model was used to compute the corresponding value of Lc that was obtained by solving the third order polynomial: 4λ^3^ + ωλ^2^ −1 = 0, where λ = 1 − D/Lc and ω = 4 F(D,Lc)/α − 3 and α = kbT/Lp. Each portion of the F-D curve was fitted perfectly by the WLC model^38^. For details please see Maity *et al*.^36^.

### Data availability

Data are available from the corresponding authors upon request.

## Electronic supplementary material

Supplementary Materials
