# Supplementary material for: The gating mechanism in cyclic nucleotide-gated ion channels
Source: Sci Rep. 2018 Jan 8;8:45. doi: 10.1038/s41598-017-18499-0 (PMC5758780; doi:10.1038/s41598-017-18499-0)
Supplement: Supplementary file 1 — Supplementary Materials [file 41598_2017_18499_MOESM1_ESM.pdf]

Supplementary Information

The gating mechanism in cyclic nucleotide-gated ion channels

Monica Mazzolini[1,#,*] Manuel Arcangeletti[1,#], Arin Marchesi[2,#], Luisa MR Napolitano[1,3], Debora Grosa[1], Sourav Maity[1], Claudio Anselmi[4] and Vincent Torre[1,*]

[1] International School for Advanced Studies, Trieste 34136, Italy;
[2] INSERM U1006, Aix-Marseille Université, Parc Scientifique et Technologique de Luminy, Marseille 13009, France.
[3] Structural Biology Laboratory, Elettra-Sincrotrone Trieste S.C.p.A., Basovizza, Trieste 34149, Italy;
[4] National Heart, Lung and Blood Institute, National Institutes of Health, Bethesda, Maryland 20892, USA

[#] Co-first author
* Corresponding authors
Lead Contact: Vincent Torre; e-mail: torre@sissa.it,
Additional Corresponding Author: Monica Mazzolini; mazzolin@sissa.it.

Supplementary Table 1

```
                                R297                         F317  A322 I323  W330                      Y347 V348 L351 L356
CNGA1_B.Taurus       TRTNYPNIFRISNLVMYIIIIIHWNACVYFSISKAIGFGNDTWVYPDVNDPD-----FGRLARKYVYSLYWSTL  356
CNGA1_Mouse          TRTNYPNIFRISNLVMYIVIIIHWNACVYYSISKAIGFGNDTWVYPDVNDPE-----FGRLARKYVYSLYWSTL  349
CNGA1_Rat            TRTNYPNIFRISNLVMYIVIIIHWNACVYYSISKAIGFGNDTWVYPDVNDPE-----FGRLARKYVYSLYWSTL  350
CNGA1_Human          TRTNYPNIFRISNLVMYIVIIIHWNACVFYSISKAIGFGNDTWVYPDINDPE-----FGRLARKYVYSLYWSTL  427
CNGA_X.Laevis        TRTNYPNIFRISNLIMYIVIIIHWNACVYYSISKAIGFGEDTWVYPNTTDPE-----YGRLARKYVYSLYWSTL  361
TAX-4_C.Elegans      TRSSMPNAFRVVVVVWYIVIIIHWNACLYFWISEWIGLGTDAWVYGHLNKQSLPDDITDTLLRRYVYSFYWSTL  372
CNGA_D.Melanogaster  TATGYPNAFRICKVVLAILVLIHWNACMYFAISYEIGFSSDSWVYN-LNGTR-----NNTLQRQYIYSFYWSTL  308
CNGB_D.Melanogaster  TATGYPNAFRICKVVLAILVLIHWNACMYFAISYEIGFSSDSWVYN-LNGTR-----NNTLQRQYIYSFYWSTL  483
CNGA4_Human          TRTAYPNAFRIAKLMLYIFVVIHWNSCLYFALSRYLGFGRDAWVYPDPAQPG-----FERLRRQYLYSFYFSTL  227
CNGA4_Rat            TRTAYPNAFRIAKLMLYIFVVIHWNSCLYFALSRYLGFGRDAWVYPDPAQPG-----FERLRRQYLYSFYFSTL  227
CNGA3_Rat            TRTNYPNVFRIGNLVLYTLIIIHWNACIYFAISKFIGFGTDSWVYPNTSKPE-----YGRLSRKYIYSLYWSTL  282
CNGA3_Mouse          TRTNYPNVFRIGNLVLYTLIIIHWNACIYFAISKFIGFGTDSWVYPNTSKPE-----YARLSRKYIYSLYWSTL  340
CNGA3_B.Taurus       TRTNYPNMFRIGNLVLYILIIIHWNACIYFAISKFIGFGTDSWVYPNVSNPE-----YGRLSRKYIYSLYWSTL  380
CNGA3_Human          TRTNYPNMFRIGNLVLYILIIIHWNACIYFAISKFIGFGTDSWVYPNISIPE-----HGRLSRKYIYSLYWSTL  361
CNGB1_D.Rerio        TRTSYPNLFRICNLVLYILVIIHWNACIYFAISKSLGFGSDQWVYPNISDPK-----FGTLTRGYVYCLYWSTL  337
CNGA2_Mouse          TRTSYPNIFRISNLVLYILVIIHWNACIYYAISKSIGFGVDTWVYPNITDPE-----YGYLAREYIYCLYWSTL  335
CNGB1_Rat            TRTSYPNIFRISNLVLYILVIIHWNACIYYVISKSIGFGVDTWVYPNITDPE-----YGYLAREYIYCLYWSTL  335
CNGA2_Human          TRTNYPNIFRISNLVLYILVIIHWNACIYYAISKSIGFGVDTWVYPNITDPE-----YGYLAREYIYCLYWSTL  333
CNGA2_B.Taurus       TRTSYPNIFRISNLILYILIIIHWNACIYYAISKSIGFGVDTWVYPNITDPE-----YGYLSREYIYCLYWSTL  333
CNGB1_Human          SILSKAYVYRVIRTTAYLLYSLHLNSCLYYWASAYQGLGSTHWVYDGV-------------GNSYIRCYYFAVK  841
CNGB1_B.Taurus       SILSKAYVYRVIRTTAYLLYSLHLNSCLYYWASAYEGLGSTHWVYDGV-------------GNSYIRCYYWAVK  955
CNGB1_Mouse          SIMDKAYVYRVIRTTGYLLFLLHINACVYYWASDYEGIGSTKWVYNGE-------------GNKYLRCFYWAVR  398
CNGB3_Human          SIMDKAYIYRVIRTTGYLLFILHINACVYYWASNYEGIGTTRWVYDGE-------------GNEYLRCYYWAVR  403
                                       H310 N312 Y316                                  Y347  Y349 W353 S354
```

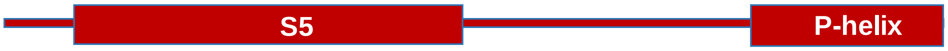


```
                                       F380                              D413
CNGA1_B.Taurus       TLTTIGETPPPVRDSEYFFVVADFLIGVLIFATIVGNIGSMISNMNAARAEFQARIDAIKQYMHFRNVSKDMEK  430
CNGA1_Mouse          TLTTIGETPPPVLDSEYIFVVVDFLIGVLIFATIVGNIGSMISNMNAARAEFQSRVDAIKQYMNFRNVSKDMEK  424
CNGA1_RAT            TLTTIGETPPPVLDSEYVFVVVDFLIGVLIFATIVGNIGSMISNMNAARAEFQSRVDAIKQYMNFRNVSKDMEK  423
CNGA1_Human          TLTTIGETPPPVRDSEYVFVVVDFLIGVLIFATIVGNIGSMISNMNAARAEFQARIDAIKQYMHFRNVSKDMEK  501
CNGA_X.Laevis        TLTTIGETPPPVQDSEFWFVVADFLVGVLIFATIVGNVGSMISNMNAARAEFQGRIDAIKQYMHFRKVSKDLEK  435
Tax-4 C.Elegans      ILTTIGEVPSPVRNIEYAFVTLDLMCGVLIFATIVGNVGSMISNMSAARTEFQNKMDGIKQYMELRKVSKQLEI  446
CNGA_D.Melanogaster  TLTTIGETPTPENDVEYLFVVADFLAGVLIFATIVGNIGSMISNMNVARVEFQNRMDGVKQYMAFRRVGHELEA  382
CNGB_D.Melanogaster  TLTTIGETPTPENDVEYLFVVADFLAGVLIFATIVGNIGSMISNMNVARVEFQNRMDGVKQYMAFRRVGHELEA  556
CNGA4_Human          ILTTVGDTPPPAREEEYLFMVGDFLLAVMGFATIMGSMSSVIYNMNTADAAFYPDHALVKKYMKLQHVNRKLER  301
CNGA4_Rat            ILTTVGDTPLPDREEEYLFMVGDFLLAVMGFATIMGSMSSVIYNMNTADAAFYPDHALVKKYMKLQHVNKRLER  301
CNGA3_Rat            TLTTIGETPPPVKDEEYLFVVIDFLVGVLIFATIVGNVGSMISNMNASRAEFQAKIDSIKQYMQFRKVTKDLET  356
CNGA3_Mouse          TLTTIGETPPPVKDEEYLFVVIDFLVGILIFATIVGNVGSMISNMNAPRVEFQAKIDSVKQYMQFRKVTKDLET  414
CNGA3_B.Taurus       TLTTIGETPPPVKDEEYLFVVIDFLVGVLIFATIVGNVGSMISNMNASRAEFQAKIDSIKQYMQFRKVTKDLET  454
CNGA3_Human          TLTTIGETPPPVKDEEYLFVVVDFLVGVLIFATIVGNVGSMISNMNASRAEFQAKIDSIKQYMQFRKVTKDLET  435
CNGB1_D.Rerio        TLTTIGEMPAPVQDEEYLFVVFDFLVGVLIFATIVGNVGAMISNMNATRAEFQGQIDAIKHYMKFRKVSKELEA  410
CNGA2_Mouse          TLTTIGETPPPVKDEEYLFVIFDFLIGVLIFATIVGNVGSMISNMNATRAEFQAKIDAVKHYMQFRKVSKDMEA  408
CNGB1_Rat            TLTTIGETPPPVKDEEYLFVIFDFLIGVLIFATIVGNVGSMISNMNATRAEFQAKIDAVKHYMQFRKVSKDMEA  408
CNGA2_Human          TLTTIGETPPPVKDEEYLFVIFDFLIGVLIFATIVGNVGSMISNMNATRAEFQAKIDAVKHYMQFRKVSKGMEA  406
CNGA2_B.Taurus       TLTTIGETPPPVKDEEYLFVIFDFLIGVLIFATIVGNVGSMISNMNATRAEFQAKIDAVKHYMQFRKVSKEMEA  406
CNGB1_Human          TLITIGGLPDPKTLFEIVFQLLNYFTGVFAFSVMIGQMRDVVGAATAGQTYYRSCMDSTVKYMNFYKIPKSVQN  914
CNGB1_B.Taurus       TLITIGGLPDPRTLFEIVFQGLNYFTGVFAFSVMIGQMRDVVGAATAGQTYYRSCMDSTVKYMNFYKIPRSVQN  1028
CNGB1_Mouse          TLITIGGLPEPQTSFEIVFQFLNFFSGVFVFSSLIGQMRDVIGAATANQNYFQACMDHIIAYMNKYSIPQSVQY  471
CNGB3_Human          TLITIGGLPEPQTLFEIVFQLLNFFSGVFVFSSLIGQMRDVIGAATANQNYFRACMDDTIAYMNNYSIPKLVQK  476
```

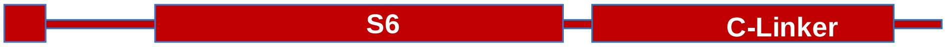

Multiple Sequence Alignment of CNG channels. Extended multiple alignment of 23 eukaryotic CNG channel sequences carried out using the multiple sequence alignment program MUSCLE[1]. Residues that are conserved in more than 70% of the eukaryotic sequences are highlighted in cyan. R297, F317, A322, I323, W330, V348, L351, L356, F380 and D413 residues are highlighted in violet. H310,N312,Y316,,Y349,W353 and S354 (Figure 5C) are highlighted in green. The following groups of amino acid residues are considered similar: Asp/Glu, Lys/Arg, Phe/Tyr, Ser/Thr, Gly/Ala, Val/Leu/Ile/Met. The predicted secondary structure is shown at the bottom. This alignment is the basis for the web-logo analysis using an open source program available at http://weblogo.berkeley.edu[2].

Supplementary Fig. 1

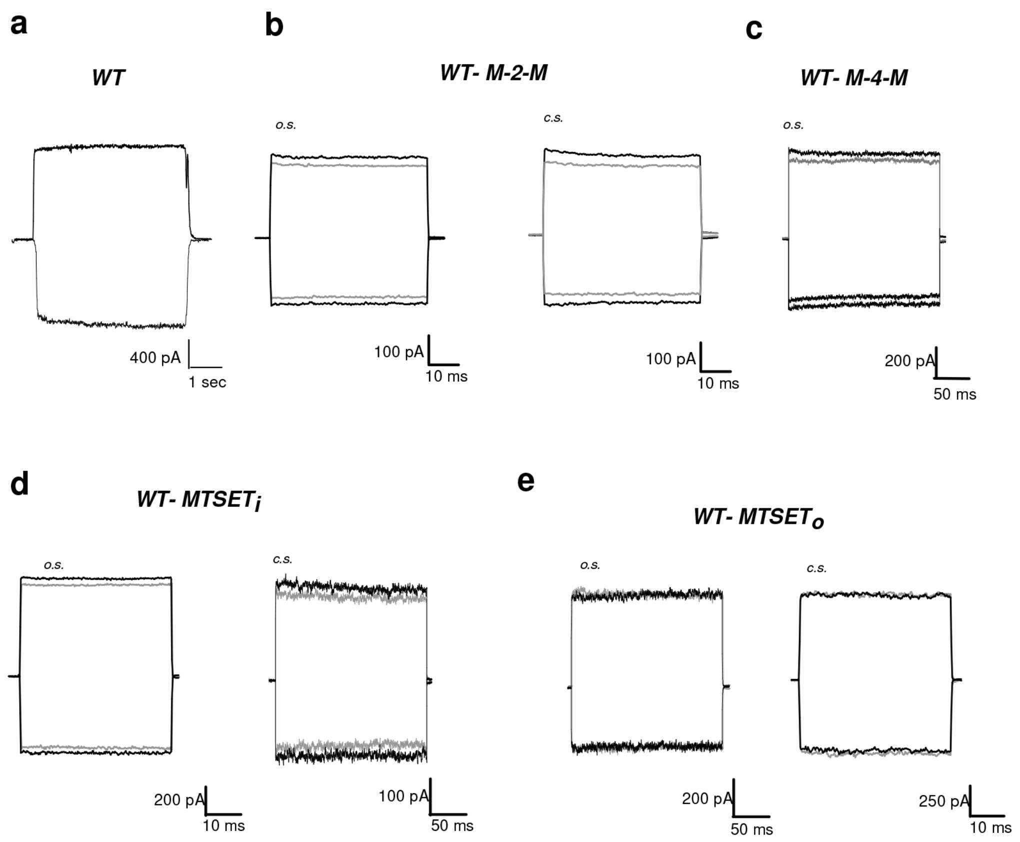

The effect of sulphydril reagents on WT CNGA1 channels. (a) Current recording evoked by 1 mM cGMP, during voltage steps at ±60 mV in symmetrical Na^+ solution for WT CNGA1. No current inactivation is observed with a marginal voltage dependent gating. (b) cGMP-activated currents at ±60 mV for WT CNGA1 channels before (black lines) and after (grey lines) 2 minutes application of 100 μM MTS-2-MTS in the open (o.s.) and closed (c.s.) state. (c) as in (B) but for 100 μM MTS-4-MTS in the open state (o.s.). (d) as in (b) but for an application of 2.5 mM MTSET to the intracellular side ($MTSET_i$). (e) the same as in (d) but with MTSET added to the extracellular side ($MTSET_o$).

Supplementary Fig.2

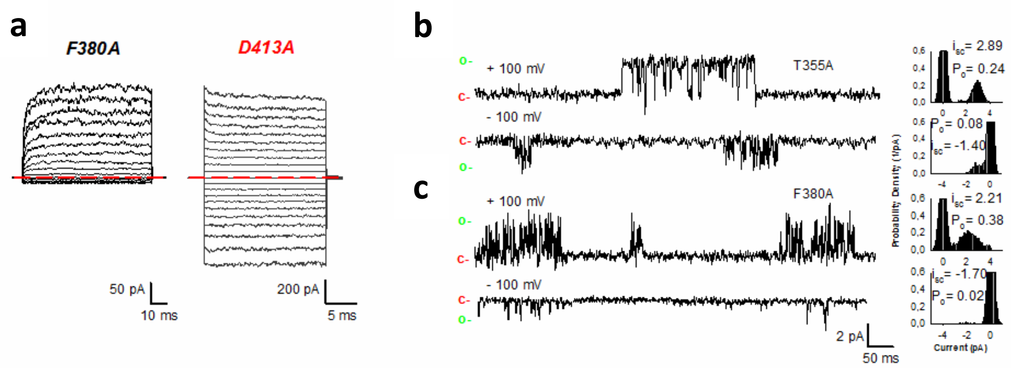

Electrical properties of mutant channels following inactivation. (a) Examples of macroscopic I/V relations obtained 2 minutes after the addition of 1 mM cGMP, i.e. after completion of inactivation, for the two types of mutant channels (F380A, left panel and D413A, right panel). Current recordings elicited by voltage steps from −200 to +200 mV (ΔV = 20 mV). (b-c) Single-channel recordings at + 100 and - 100 mV for mutant channels T355A (b) and F380A (c). Amplitude histograms from recordings in (b) and (c) are shown at the right (grey area). Solid black lines represent a two-components Gaussian fit to the histograms. The analysis of the amplitude histograms (n=3) shows the amplitude of single channel currents is very similar at positive and negative voltages and that the open probability is about 2-10 times lower at negative than at positive voltages. Therefore the inactivation is caused by a time dependent decrease of the open probability (see also Bucossi et al.[3]).

Supplementary Fig.3

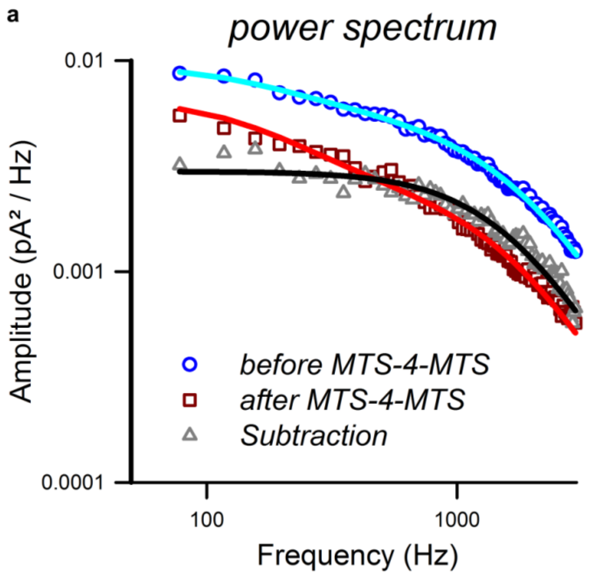

Power spectrum of cGMP activated currents in the double mutant channels D413C_R297C - Power spectra of current fluctuations in the presence of 1 mM cGMP, before (empty blue circles) and after (empty brown squares) the application of 100 µM MTS-4-MTS, at +40 mV. Subtracted power spectrum shown with grey empty triangles. Current recordings were filtered at 20 kHz and sampled at 60 kHz. Power spectra were computed as described by Sesti et al; 1994[4]. Power spectra for recordings obtained before and after the application of 100 µM MTS-4-MTS were fitted with the sum of three Lorentzian functions with f1, f2 and f3 values of 177, 524 and 1600 Hz respectively and with S1(0), S2(0) and S3(0) values of 3.9 x 10-3, 5.2 x 10-4 and 5.1 x 10-3 pA2/Hz and 3.9 x 10-3, 5.2 x 10-4 and 2.1 x 10-3 pA2/Hz for current recordings before and after the application of MTS-4-MTS respectively. Power spectrum for the subtraction curve was fitted with a single Lorentzian function with an f value of 1600 Hz and an S(0) value of 3 x 10-3 pA2/Hz. Analysis of the power spectrum of these fluctuations shows that the Lorentzian component with a cut-off frequency around 1600 Hz is reduced when the electrostatic interactions between D413 and R297 are replaced by a S-S bond between residues in the same locations.

Supplementary Fig. 4

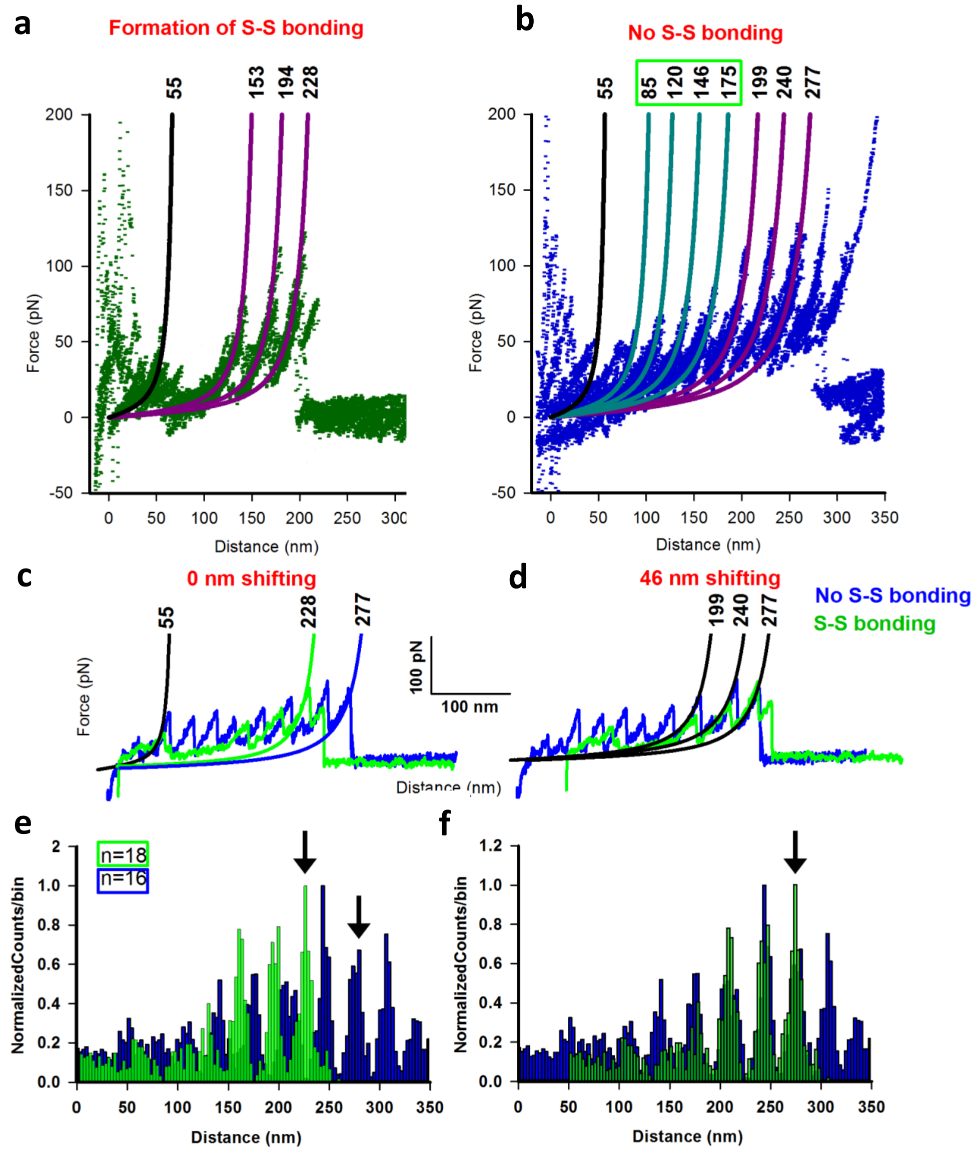

SMFS experiments on double mutant channel D413C_R297C. (a) Unfolding of a single subunit of the double mutant channels with an unfolded length of about 228 nm, associated to the formation of an S-S bond between exogenous cysteines. Continuous lines represent the WLC fitting of the corresponding peaks, and the numbers are the corresponding values of Lc. Black lines are the fitting of the peaks that correspond to 55 nm, similar to what is observed in the CNGA1 at open state; violet lines are the fitting of the peaks representing the unfolding of the transmembrane domains S4-S3 and S2-S1 helices. (b) unfolding pattern of the mutant channel with an unfolded length >277 nm (in Lc), similar to the unfolding pattern that is observed in the WT CNGA1 channel in the open state, denoting the absence of the formation of an S-S bond between exogenous cysteines. Continuous lines represent the WLC fitting of the corresponding peaks and the numbers are the corresponding Lc values, in agreement to what is observed for CNGA1 channels in the open state. Cyan lines and corresponding numbers (in green box) represent the peaks that are missing during the unfolding of the mutant channel when a S-S bond is formed (A), violet lines represent the same peaks present in a, but shifted of 46 nm in Lc. (c-d) Comparison of single traces from panel a (green trace) and panel b (blue trace) with zero shifting, i.e. only the 55 nm peak is superimposed and with 46 nm shifting, i.e. the last three peaks at 199, 240, 277 nm are superimposed. (e-f) **C**umulative Lc histogram obtained as in Maity et al., 2015[5].

Supplementary Fig. 5

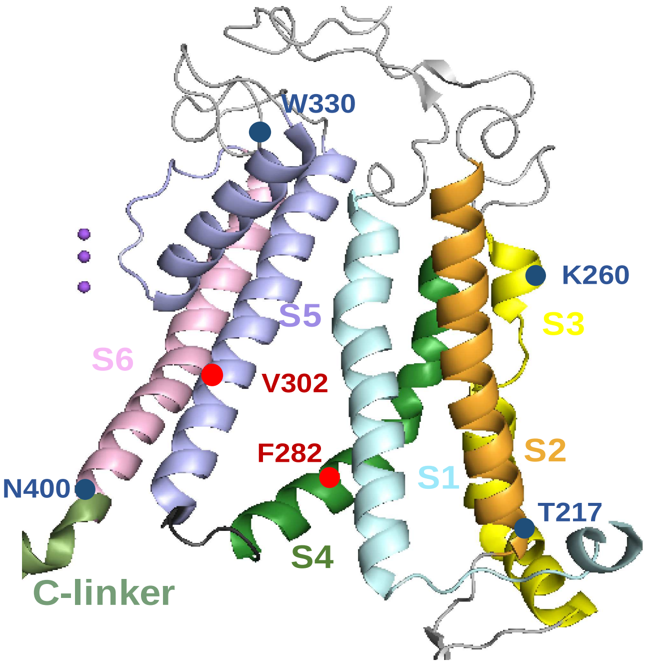

Position of the residues shown in Figure 3A on the Tax-4 protomer structure. We mapped the amino-acid residues shown in Figure 3A on the Tax-4 protomer structure (PDB ID: 5H3O). Different regions (S1-S6 and C-linker) are shown in different colors.

Supplementary Fig. 6

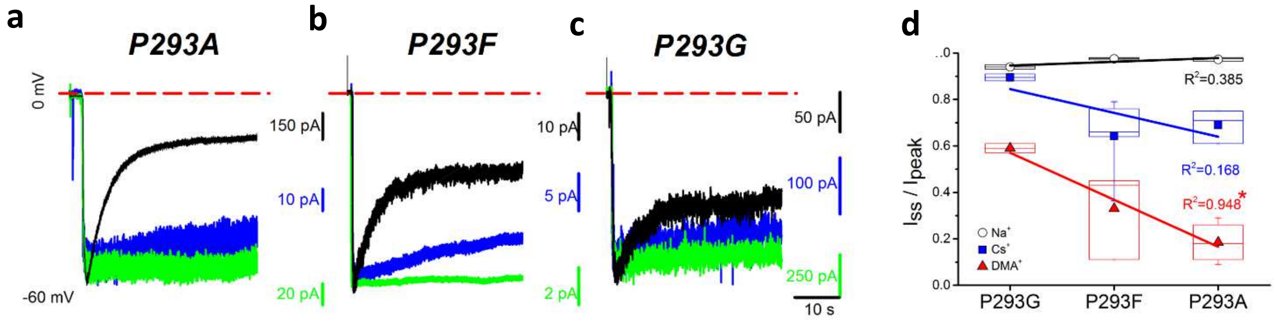

Electrical recordings from P293 mutant channels. (a,b,c) cGMP-activated currents for the mutant channels P293A, P293F and P293G respectively, in the presence of 1 mM cGMP at -60 mV and in the presence of different symmetrical ion solutions (Na^+-green, Cs^+-blue, DMA^+-black). (d) box plot of the residual currents after inactivation versus the ion species for the three mutant channels. The horizontal line within each box indicates the median of the data; boxes show the twenty-fifth and seventy-fifth percentiles of the data; whiskers show the fifth and ninety-fifth percentiles of the data. Linear regression analysis indicates that the fractional steady state current (I_{ss}/I_{peak}) correlate with the helical propensity of the amino acid being replaced to Proline in position 297. For the linear regression analysis the mean for each data set was used (symbols within each pox plot). Mutant channels were ordered on the x-axis according to their helix-propensity energy profiles (ref. 6; glycine > phenylalanine > alanine). (*) indicates a statistical significance with $P < 0.05$.

Supplementary Fig. 7

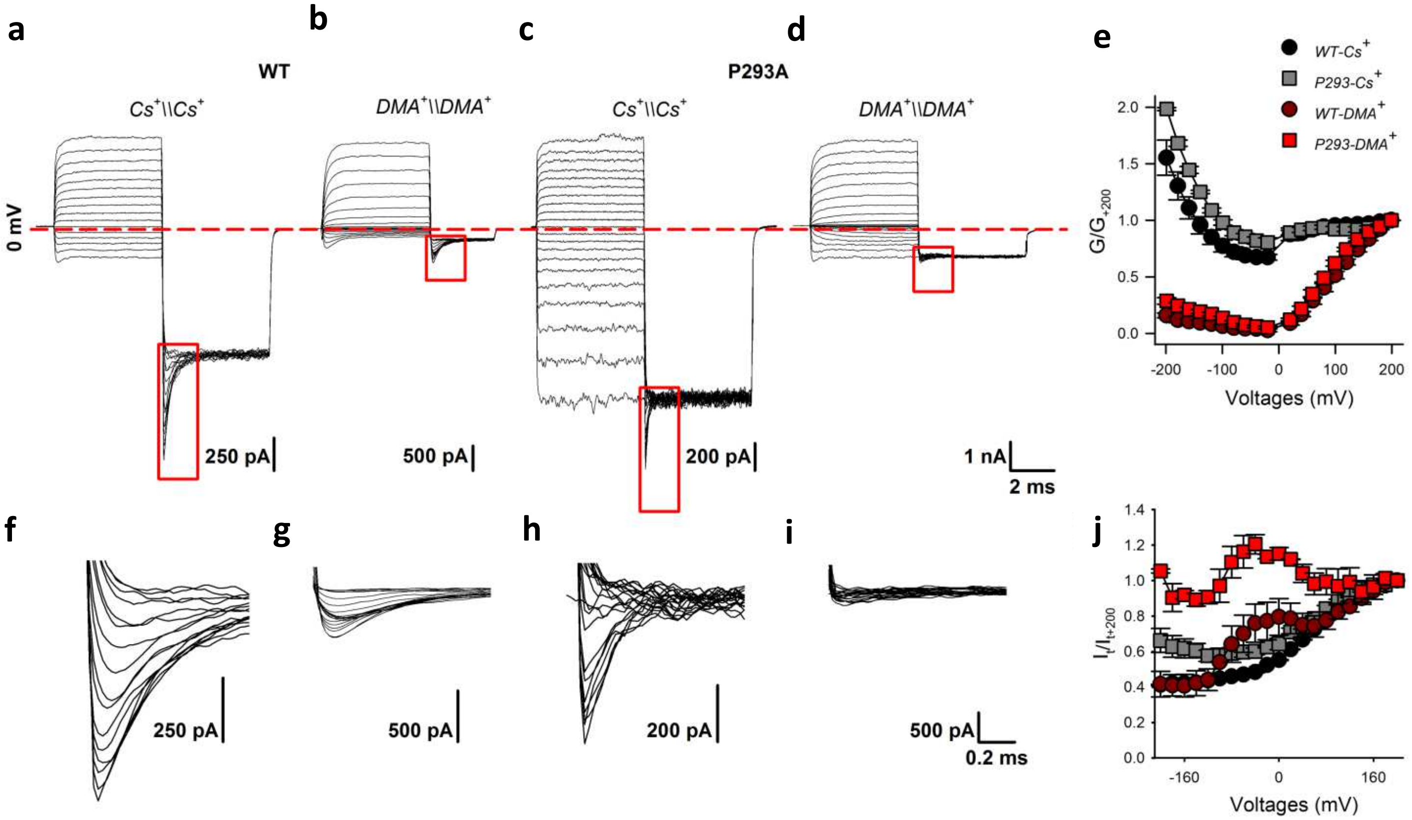

Comparison of electrical properties of WT and P293A mutant channels. (a-d) Macroscopic currents recorded from excised patches in symmetrical solutions of Cs+ (a,c) and DMA+ (b,d) for WT (a,b) and P293A (c,d) mutant channels with 1 mM cGMP in the intracellular medium. Leak and capacitive components were removed subtracting from the cGMP-activated current those recordings obtained in response to the same voltage protocol, but without cGMP. The voltage commands were stepped from a holding potential of 0 mV to prepulses varying between −100 and +200 mV in 20 mV steps. At the end, the voltage command was moved to −200 mV for 5 msec in order to elicit tail currents It (V). Red broken line indicates 0 current level. (f-i) Enlargement of tail currents (boxed areas in A-D). Current recordings were filtered at 10 kHz and sampled at 50 kHz to resolve rapid transients. (e) Dependence of G/G+200 on V in symmetrical solutions of Cs+ (black and grey) and DMA+ (red and dark red) for WT (circles) and P293A (squares) mutant channels. (j) Estimation of Po/Po_max from tail currents. Po/Po_max was estimated as It/It+200; symbols and colors are the same as in (e).

Supplementary Table 2

a

Unpaired two-tail T-test (Steady-State Currents at +60 vs -60mV), referred to Figure 1D							
**Mutant**	T355A	L356A	E363A	T364A	F380A	R297A	D413A
**n**	4;4	6;6	4;6	4;4	6;4	2;2	4;4
**DoF**	6	10	6	6	10	2	4
**t**	7,911	2,274	6,881	-5,541	5,652	-0,0967	1,084
**P**	<0,001†	0,0463	<0,001	0,00146	<0,001	0,932†	0,32
**Stat. Significance**	***	*	***	**	***	ns	ns

b

Unpaired two-tail T-test (Tau at +60 vs -60mV), referred to Figure 1E							
**Mutant**	T355A	L356A	E363A	T364A	F380A	R297A	D413A
**n**	4;4	6;6	4;6	4;4	6;4	2;2	4;4
**DoF**	6	6	8	6	10	2	6
**t**	12,981	-24,02	19,01	41,021	14,985	1,172	1,67
**P**	<0,001	<0,001	<0,001	<0,001	<0,001	0,362	0,146
**Stat. Significance**	***	***	***	***	***	ns	ns

c

Unpaired two-tail T-test (Open vs Closed State), referred to Figure 4I							
**Mutant**	**R297C**	**I298C**	**S299C**	**N300C**	**L301C**	**V302C**	**M303C**
**n**	4;5	4;5	3;3	4;5	8;8	3;3	3;3
**DoF**	7	7	4	7	14	4	4
**t**	-7,529	-1,988	1,005	0,325	-1,36	-0,966	0,794
**P**	<0,001	0,0871	0,372	0,754†	0,195	0,389	0,472
**Stat. Significance**	***	n.s.	n.s.	n.s.	n.s.	n.s.	n.s.
**Mutant**	**Y304C**	**I305C**	**I306C**	**I308C**	**WT**	**V320C**	**M323C**
**n**	7;3	4;3	6;6	3;4	4;5	3;4	3;3
**DoF**	8	5	10	5	7	5	4
**t**	-2,649	-0,972	-0,48	-0,681	1,268	0,672	1,062
**P**	0,0293	0,376	0,642	0,526†	0,245	0,531	0,348
**Stat. Significance**	*	n.s.	n.s.	n.s.	n.s.	n.s.	n.s.

One-way ANOVA (Open state), referred to Figure 4I

DoF	13,45
F	6,783
P	<0.001
Stat. Significance	***

Pairwise Comparisons vs WT (Holm-Sidak)

Mutant	R297C	I298C	S299C	N300C	L301C	V302C	M303C
n	4	4	3	4	8	3	3
P	0,884	0,866	0,903	0,906	0,98	0,46	0,863
Stat. Significance	ns	ns	ns	ns	ns	ns	ns

Mutant	Y304C	I305C	I306C	I308C	V320C	M323C
n	7	4	6	3	3	3
P	<0.001	0,88	0,945	0,403	0,907	0,811
Stat. Significance	***	ns	ns	ns	ns	ns

One-way ANOVA (Closed state), referred to Figure 4I

DoF	13,46
F	8,181
P	<0.001
Stat. Significance	***

Pairwise Comparisons vs WT (Holm-Sidak)

Mutant	R297C	I298C	S299C	N300C	L301C	V302C	M303C
n	5	5	3	5	8	3	3
P	<0,001	0,624	0,162	0,802	0,307	0,75	0,409
Stat. Significance	***	ns	ns	ns	ns	ns	ns

Mutant	Y304C	I305C	I306C	I308C	V320C	M323C
n	3	3	6	4	4	3
P	0,791	0,785	0,765	0,174	0,842	0,404
Stat. Significance	ns	ns	ns	ns	ns	ns

d

Unpaired two-tail T-test (Open vs Closed) referred to Figure 4J						
Mutant	WT	A322C	I323C	N327C	V348C	L351C
n	3;3	7;3	4;5	3;3	7;7	4;4
DoF	4	8	7	4	12	6
t	0,538	-3,668^	-0,399	-0,72	-4,544	-3,109
P	0,619	0,00633	0,702	0,511	<0.001	0,0209
Stat. Significance	n.s.	**	n.s.	n.s.	***	*

One-way ANOVA (Open state), referred to Figure 4J	
DoF	5,22
F	23,688
P	<0.001
Stat. Significance	***

Pairwise Comparisons vs WT (Holm-Sidak)					
Mutant	A322C	I323C	N327C	V348C	L351C
n	7	4	3	7	4
P	0,01	<0.01	0,79	<0.01	0,945
Stat. Significance	*	**	n.s.	**	n.s.

One-way ANOVA (Closed state), referred to Figure 4J	
DoF	5,19
F	41,462
P	<0.001
Stat. Significance	***

Pairwise Comparisons vs WT (Holm-Sidak)					
Mutant	A322C	I323C	N327C	V348C	L351C
n	3	5	3	7	4
P	0,41	<0.001	0,57	<0.001	<0.01
Stat. Significance	n.s.	***	n.s.	***	**

e

Unpaired two-tail T-test (Open vs Closed) referred to Figure 6D

Mutant	F375C	V376C	V377C	A378C	D379C	F380C	I382C	G383C
n	3;3	3;3	3;4	3;3	3;3	6;6	3;3	6;4
DoF	4	4	5	4	4	10	4	8
t	-0,114	-0,378	-9,371	-7,632	0,981	-7,407	6,877	-2,278
P	0,915^	0,725	<0,001	<0,01	0,382	<0,001†	<0,01†	0,052
Stat. Significance	n.s.	n.s.	***	**	n.s.	***	**	n.s.
**Mutant**	**L385C**	**I386C**	**I390C**	**V391C**	**G392C**	**S399C**	**N402C**	**A406C**
n	4;5	4;4	6;2	3;3	5;5	6;8	3;3	4;4
DoF	7	6	6	4	8	11	4	6
t	11,569	-3,45	-0,465	25,158	2,228	-2,147	-27,762	2,438
P	<0,001†	0,0136	0,658^	<0,001	0,0565	0,0549	<0,001	0,0506
Stat. Significance	***	*	ns	***	ns	ns	***	ns
**Mutant**	**Q409C**	**D413C**	**A414C**	**D416C**	**Q417C**	**H420C**		
n	5;5	4;4	3;4	4;4	4;4	3;3		
DoF	8	6	5	6	6	4		
t	-9,907	6,212	0,761	4,775	5,945	-2,689		
P	<0,001	<0,001	0,481	<0,01	<0,01	0,0547		
Stat. Significance	***	***	ns	**	**	ns		

One-way ANOVA (Open state), referred to Figure 6D

DoF	22,68
F	14,029
P	<0.001
Stat. Significance	***

Pairwise Comparisons vs WT (Holm-Sidak)

Mutant	F375C	V376C	V377C	A378C	D379C	F380C	I382C	G383C
n	3	3	3	3	3	6	3	6
P	0,544	0,561	0,062	0,014	0,507	<0,001	0,357	0,047
Stat. Significance	ns	ns	ns	*	ns	***	ns	*

Mutant	L385C	I386C	I390C	V391C	G392C	S399C	N402C	A406C
n	4	4	6	3	5	6	3	4
P	0,56	0,584	<0,001	<0,001	0,398	0,148	0,543	0,622
Stat. Significance	ns	ns	***	***	ns	ns	ns	ns

Mutant	Q409C	D413C	A414C	D416C	Q417C	H420C
n	5	4	3	4	4	3
P	0,292	<0,001	0,473	0,64	0,554	0,604
Stat. Significance	ns	***	ns	ns	ns	ns

One-way ANOVA (Closed state), referred to Figure 6D

DoF	22,69
F	16,986
P	<0,001
Stat. Significance	***

Pairwise Comparisons vs WT (Holm-Sidak)

Mutant	F375C	V376C	V377C	A378C	D379C	F380C	I382C	G383C
n	3	3	4	3	3	6	3	4
P	0,848	0,824	0,873	0,835	0,697	0,717	<0,001	0,728
Stat. Significance	ns	ns	ns	ns	ns	ns	***	ns

Mutant	L385C	I386C	I390C	V391C	G392C	S399C	N402C	A406C
n	5	4	2	3	5	8	3	4
P	<0,001	0,988	0,081	0,714	0,883	0,561	0,989	0,826
Stat. Significance	***	ns	ns	ns	ns	ns	ns	ns

Mutant	Q409C	D413C	A414C	D416C	Q417C	H420C
n	5	4	4	4	4	3
P	<0,001	<0,001	0,956	0,735	0,4	0,904
Stat. Significance	***	***	ns	ns	ns	ns

f

Unpaired two-tail T-test (Open vs Closed) referred to Figure 7C				
**Mutant**	**Q409C**	**A414C**	**Q417C**	**WT**
**n**	3;5	6;5	2;2	2;2
**DoF**	9	2	2	6
**t**	0,33	-0,736	-6,57	-2,724
**P**	0,749	0,539	0,0224†	0,0345†
**Stat. Significance**	ns	ns	*	*

One-way ANOVA (Open state), referred to Figure 7C	
**DoF**	3,9
**F**	108,831
**P**	<0,001
**Stat. Significance**	***

Pairwise Comparisons vs WT (Holm-Sidak)			
**Mutant**	**Q409C**	**A414C**	**Q417C**
**n**	6	2	2
**P**	<0,001	0,894	0,695
**Stat. Significance**	***	ns	ns

One-way ANOVA (Closed state), referred to Figure 7C	
**DoF**	5,17
**F**	13,753
**P**	<0,001
**Stat. Significance**	***

Pairwise Comparisons vs WT (Holm-Sidak)				
**Mutant**	**A406**	**Q409C**	**A414C**	**Q417C**
**n**	5	5	2	2
**P**	0,396	<0,001	0,623	0,044
**Stat. Significance**	ns	***	ns	*

Statistical analysis. Tables (a), (b), (c), (d), (e) and (f) refer to the results from the indicated statistical tests performed on data presented in Fig. 1d, 1e, 4i, 4j, 6d and 7b of the main text, respectively. Statistical significance of voltage-dependent inactivation versus non voltage-dependent inactivation was established using unpaired two-tailed t-test (a,b). Statistical significance of the MTSET/M2M state-dependent effect (MTSET/M2M applied in the presence versus absence of cGMP) for each mutant channel was assessed using unpaired two-tailed t-test (c-f). Statistical significance of the MTSET effect (blockage or potentiation) among all mutant channels was established by single-variable ANOVA. For pairwise comparisons with WT (control), a Holm–Sidak test was used as *post hoc* test (c-f). F indicates the F-value obtained from one-way ANOVA. t indicates the t-value obtained from t-test. DoF refers to degree of freedom. n indicates the number of patches. (^) indicates a P<0,05 in Normality test (Shapiro-Wilk). (†) indicates a P<0,05 in Equal variance test. n.s. indicates a not statistically significant P value. (*) P < 0,05; (**) P < 0,01; (***) P < 0,001.

Supplementary Fig. 8

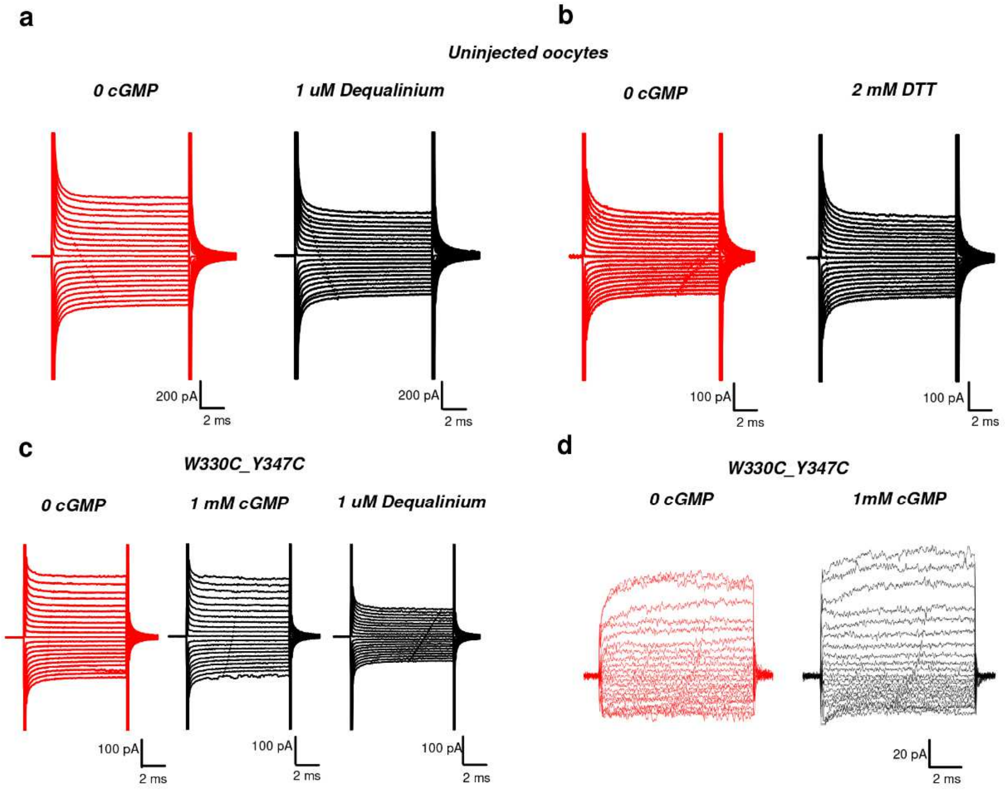

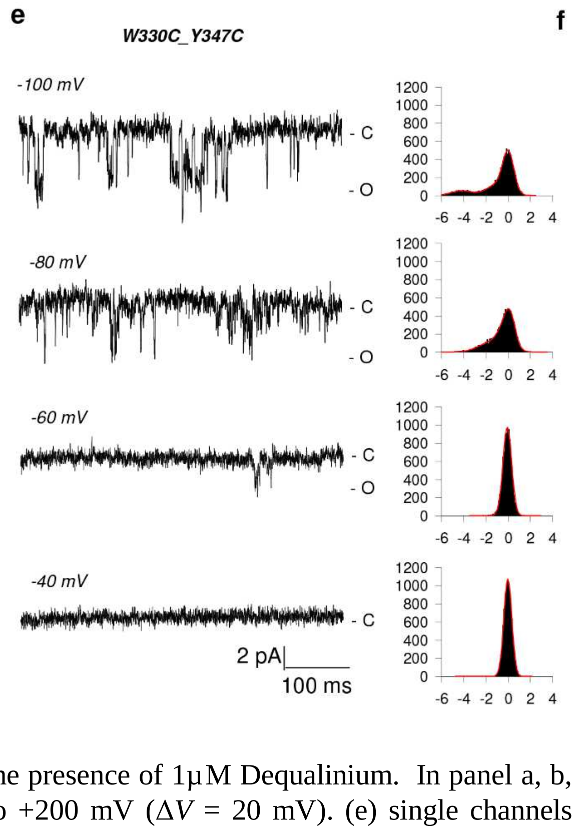

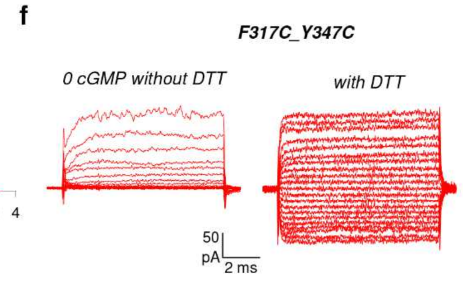

The effect of Dequalinium and DTT on membrane patches. (a,b) Macroscopic currents recorded from excised patches in symmetrical Na^+ solutions for uninjected oocytes with 1 µm Dequalinium (a) and 2 mM DTT (b) in the intracellular medium. (c) Macroscopic currents recorded from excised patches in symmetrical Na^+ solutions for double mutant channels W330C_Y347C in the absence of cGMP (left), in the presence of 1 mM cGMP (center) and with 1 µM Dequalinium (right) in the intracellular medium. (d) The same as (c) but removing the leak and capacitative components by subtracting currents in the presence of cGMP to currents without cGMP those obtained in response to the same voltage protocol but in the presence of 1µM Dequalinium. In panel a, b, c and d current recordings were elicited by voltage steps from −200 to +200 mV (ΔV = 20 mV). (e) single channels recordings obtained in the absence of cGMP for mutant channels W330C_Y347C, elicited by different voltages. Amplitude histograms are shown at the right of each trace. *Ics* indicates the estimated single-channel current amplitude. (f) In panel left current recordings obtained in the absence of cGMP (0 cGMP) for mutant channels F317C_Y347C, elicited by voltage steps from −200 to +200 mV (ΔV = 20 mV); in panel right the same but after 5 minute of the addition of 2 mM DTT.

Supplementary Fig. 9

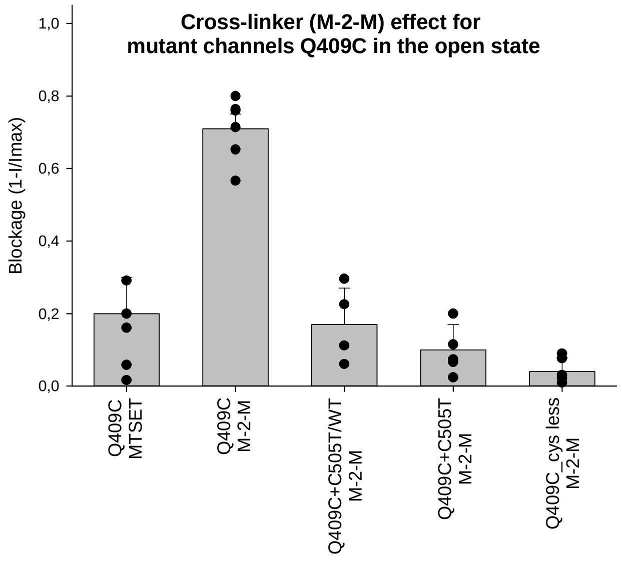

The effect of the cross-linker M-2-M on mutant channel Q409C in the open state.
Blockage by MTSET in the open state on mutant channels Q409C (n=6), blockage by M-2-M of mutant channels Q409C_C505T (n=5), of mutant channels Q409C_C505T/WT tandem (n=4) and Q409C in the cysless CNGA1 background (n=5). M-2-M blockage was abolished in the double mutant Q409C + C505T and in the tandem construct Q409C + C505T/WT as well as in mutant channels Q409C in in CNGA1 channels without endogenous cysteines[7,8].
In order to identify the molecular mechanisms underlying the open state inhibition, we analyzed the effect of 100 μM M-2-M on mutant channels Q409C when C505 was replaced with a threonine and in this case M-2-M did not inhibit the double mutant channels Q409C + C505T. Therefore, inhibition of Q409C in the open state caused by M-2-M is due to its cross-linking to 409C and C505. In order to establish whether the inhibition was a consequence of the cross-linkage between two cysteines from the same subunit or from neighboring subunits, M-2-M inhibition in the open state was analyzed in the tandem Q409C + C505T/WT. In this tandem construct, each subunit contained either 409C or C505 and the cross-linkage between 409C and the C505 can occur only between different subunits. M-2-M in the open state did not inhibit the tandem construct Q409C + C505T/WT and it is concluded that inhibition of mutant channels Q409C by M-2-M in the open state is caused by the cross-linkage of C505 with 409C of the same subunit.

References

1.Sievers, F. et al. (2014) Fast, scalable generation of high-quality protein multiple sequence alignments using Clustal Omega. Molecular Systems Biology 7, 539–539.

2.Crooks, G.E., Hon, G., Chandonia, J.-M. and Brenner, S.E. (2004) WebLogo: a sequence logo generator. Genome Res. 14, 1188–1190.

3.Bucossi, G., Eismann, E., Sesti, F., Nizzari, M., Seri, M., Kaupp, U.B., and Torre, V. (1996) Time-dependent current decline in cyclic GMP-gated bovine channels caused by point mutations in the pore region expressed in Xenopus oocytes. J Physiol. 493, 409-18.

4.Sesti, F., Straforini, M., Lamb, T.D. and Torre, V. (1994) Gating, selectivity and blockage of single channels activated by cyclic GMP in retinal rods of the tiger salamander. The Journal of Physiology 474, 203.

5.Maity, S. et al. (2015) Conformational rearrangements in the transmembrane domain of CNGA1 channels revealed by single-molecule force spectroscopy. Nat Commun 6, 7093.

6.Pace, C.N. and Scholtz, J.M. (1998) A helix propensity scale based on experimental studies of peptides and proteins. Biophys. J. 75, 422–427.

7.Nair, A.V., Anselmi, C., and Mazzolini, M. (2009) Movements of native C505 during channel gating in CNGA1 channels. Eur Biophys J. 38, 465-78.

8.Nair, A.V., Nguyen, C.H., and Mazzolini M. (2009) Conformational rearrangements in the S6 domain and C-linker during gating in CNGA1 channels. Eur Biophys J. 38, 993-1002.
